# Supplementary figures and images for: Dynamic balance between vesicle transport and microtubule growth enables neurite outgrowth
Source: PLoS Comput Biol. 2019 May 1;15(5):e1006877. doi: 10.1371/journal.pcbi.1006877 (PMC6546251; doi:10.1371/journal.pcbi.1006877)

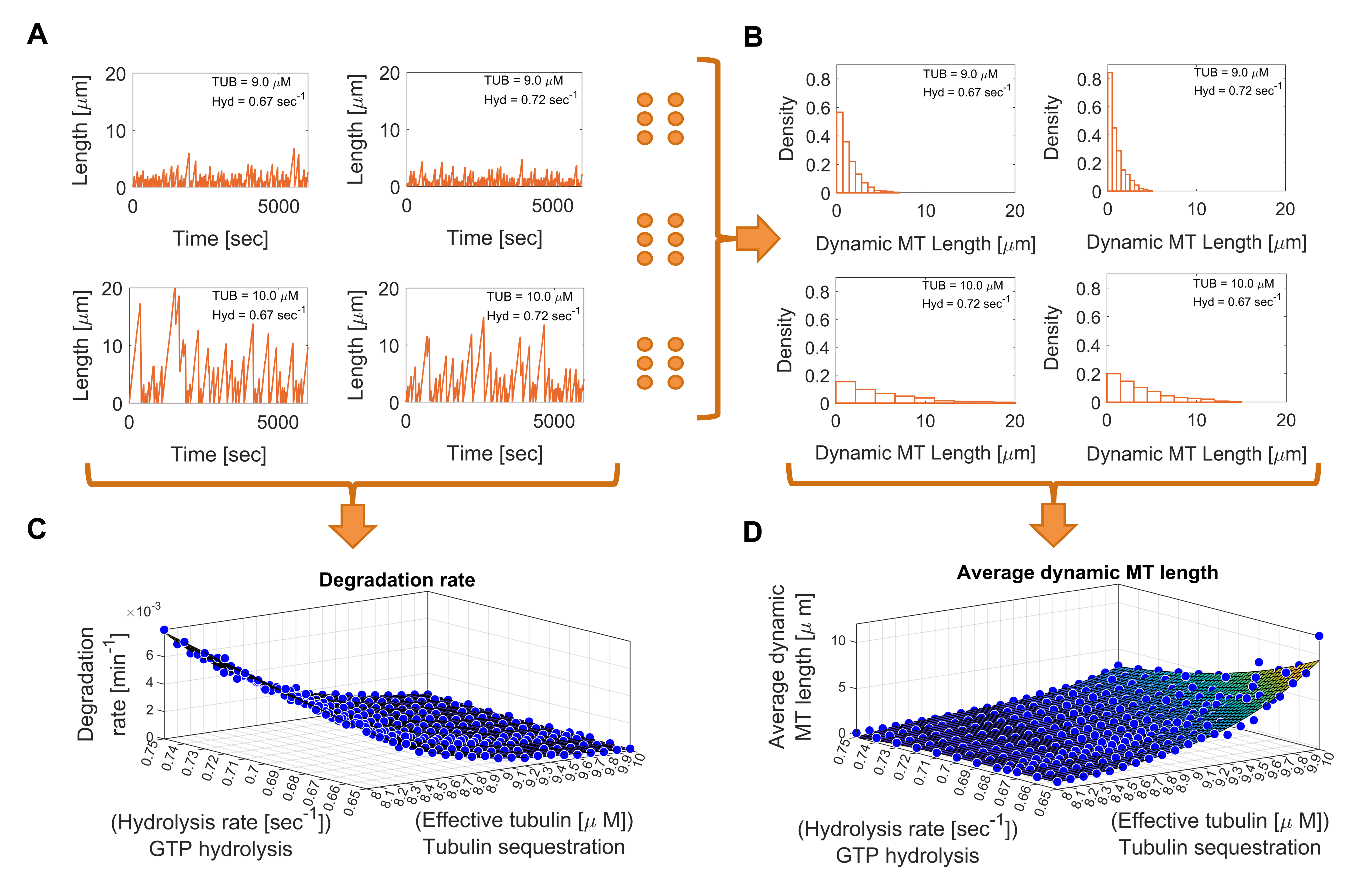

Supplement: S1 Fig — (A) Using the model by Margolin et al [35], we generated four growth profiles of single dynamic microtubules over a time period of 26,000 secs under varying effective tubulin concentrations (ranging from 8 to 10 μM) as well as varying GTP hydrolysis rates (ranging from 0.65/sec to 0.75/sec). Four example profiles are shown for (effective tubulin = 9 μM and hydrolysis rate = 0.67 sec-1, 0.72 sec-1, figures shown only initial 6000 seconds). (B) We identified the length distribution for each growth. Shown are the distribution of the four examples in (A). (C) The average length of the dynamic microtubule calculated for given range of effective tubulin concentrations, hydrolysis rates and used third order cubic polynomials to fit the data. We used all average lengths to generate a formula that describes the dependence of the average MT length on the effective tubulin concentration and the GTP hydrolysis rate. This formula was incorporated into the main model (D) To determine the degradation rate of dynamic MTs in dependence of the effective tubulin concentration and GTP hydrolysis rate, we counted how often the simulated dynamic MT of each growth profile falls below the length of 4 tubulin dimers that we defined as the threshold for complete catastrophic breakdown. We used the results to generate a formula that describes the dependence of the degradation rates on the effective tubulin concentration and the GTP hydrolysis rate. This formula was incorporated into the main model. (TIF) [file pcbi.1006877.s001.tif]

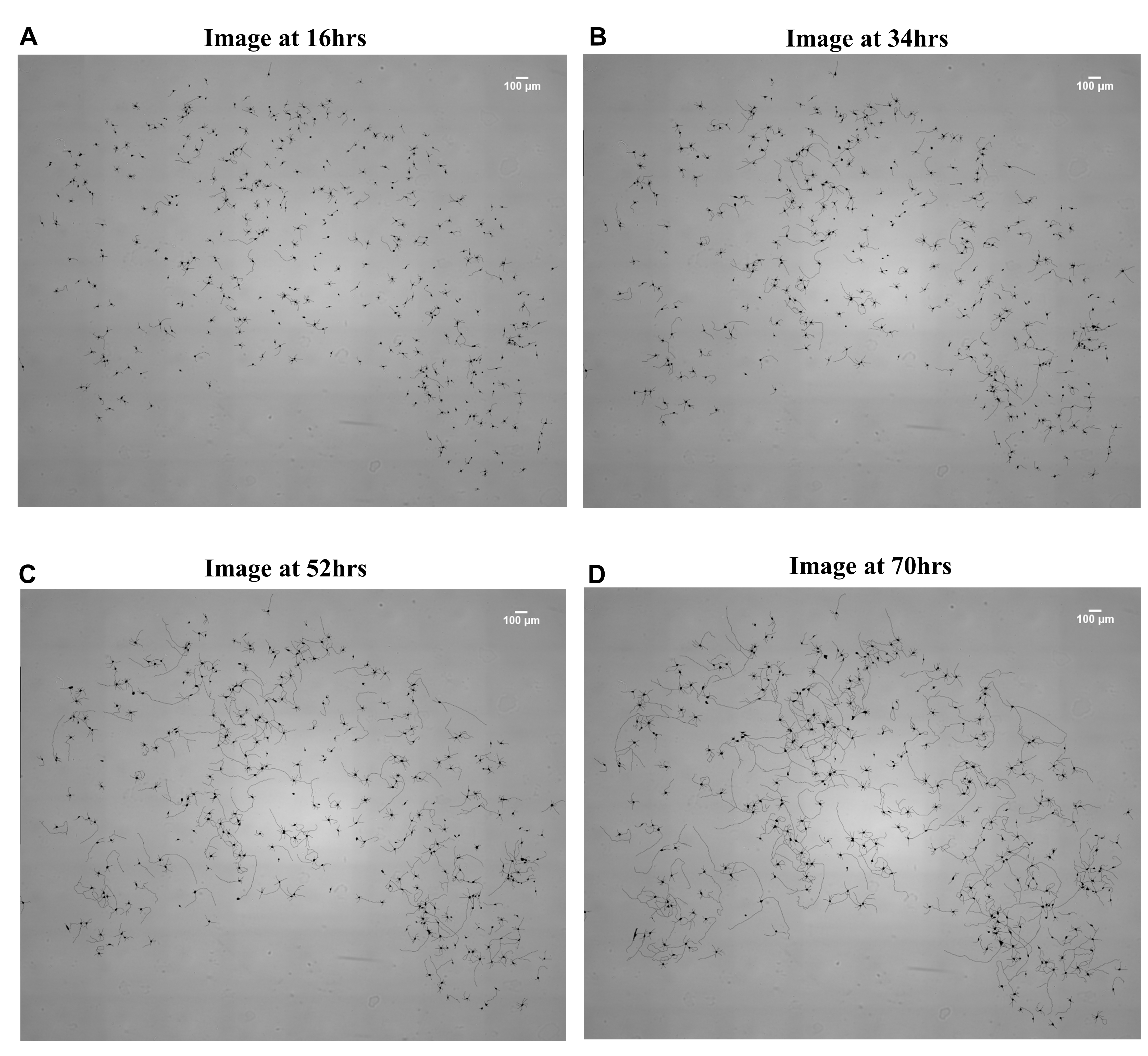

Supplement: S2 Fig — Neurons were dissected from rat cortical brain, plated on 96 well plates and incubated for 16h to allow initial growth, followed by Image acquisition every 6h up to 70h after plating. Images were pseudo colored and subjected to neurite length quantification via Metamorph. (TIF) [file pcbi.1006877.s002.tif]

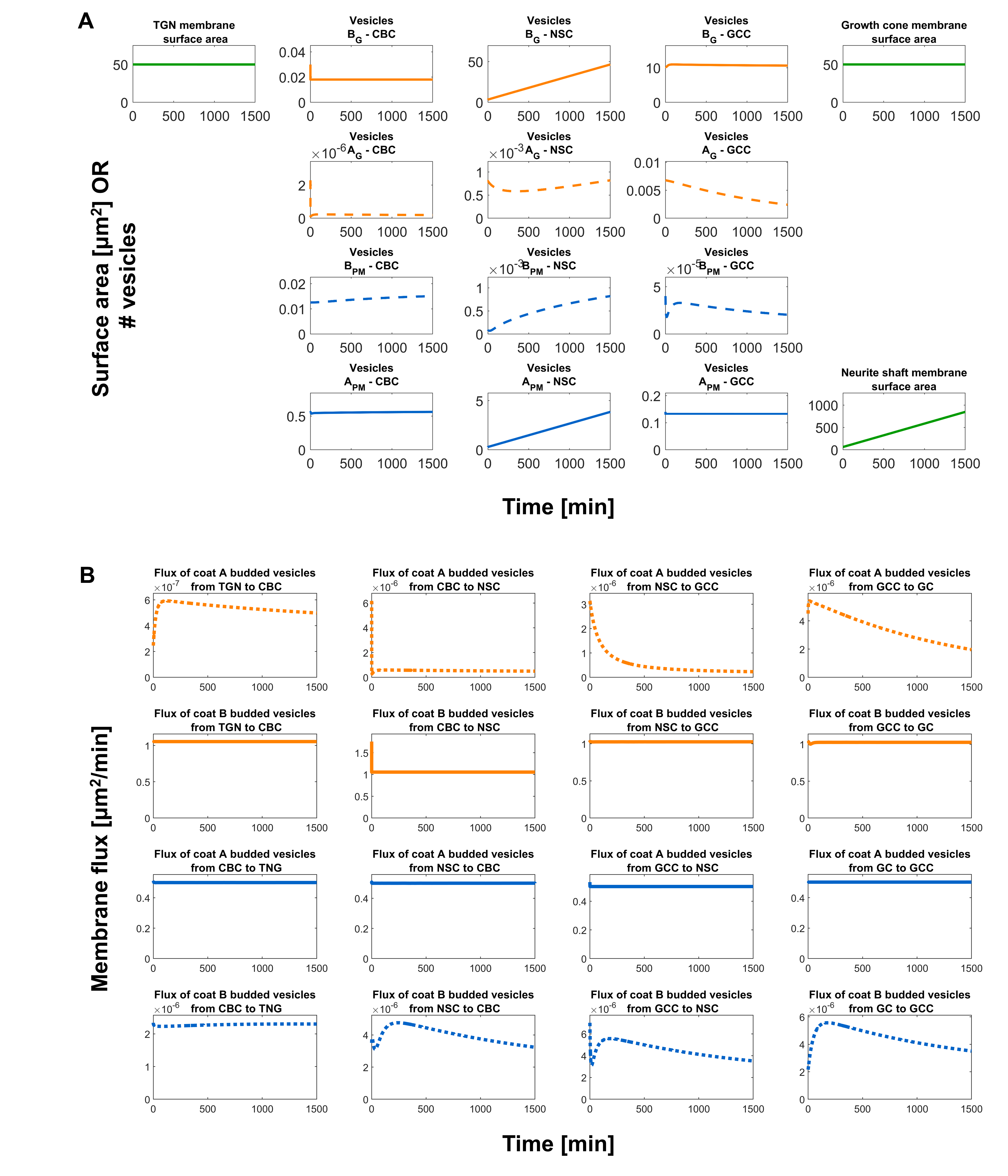

Supplement: S3 Fig — (A) At steady state all protein and membrane amounts stay constant in the different compartments, except in the neurite shaft cytoplasm (NSC) and the neurite shaft. Neurite shaft growth is facilitated by continuous membrane addition, so the membrane surface area of the neurite shaft grows over the time. The NSC grows parallel to the neurite shaft and acts as a sink for transport vesicles, thereby accumulating vesicle membrane and vesicle membrane proteins. The figures show the membrane surface areas in each compartment within each set of vesicles (in contrast to Fig 4C that shows the number of vesicles). BG refers to anterograde vesicles that bud from the TGN with the coat protein B, AG refers to anterograde vesicles that bud from the TGN with the coat A, BPM refers to retrograde vesicles that bud from the GC-PM with coat protein B and APM to retrograde vesicles that bud from the GC with coat protein A. Green lines refer to TGN or growth cone plasma membrane (GC-PM), orange lines refer to anterograde moving vesicles (BG, AG) and blue lines to retrograde moving vesicles (BPM, APM). Solid lines represent those vesicles that are mainly responsible for membrane transport in the indicated direction (i.e. BG in the anterograde direction and APM in the retrograde direction). (B) Constant membrane fluxes document steady state dynamics. (Notify that the fluxes of coat A budded vesicles from the TGN and coat B budded vesicles from the GC-PM are very low and therefore negligible). Colors and line styles are the same as in (A). (TIF) [file pcbi.1006877.s003.tif]

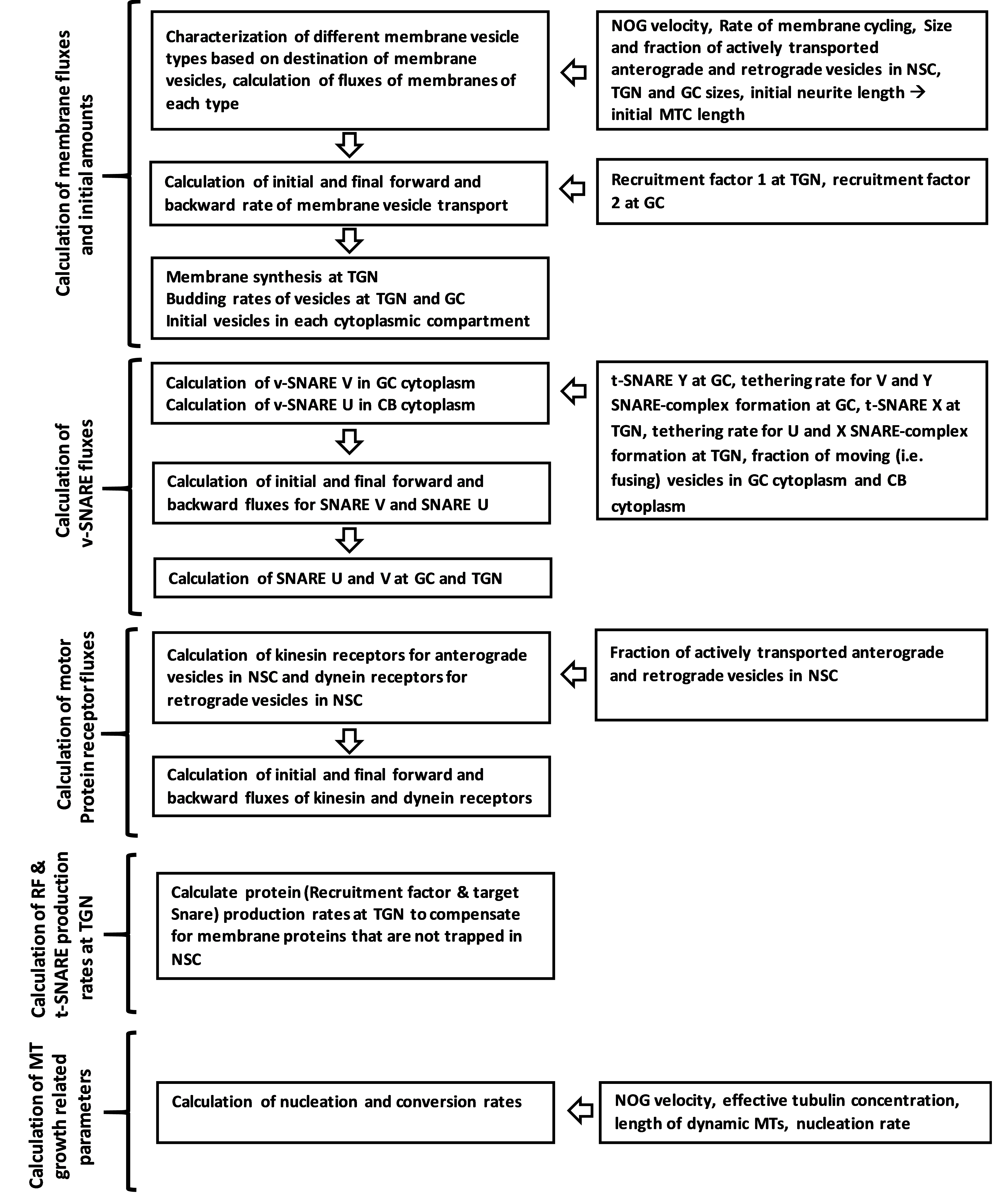

Supplement: S4 Fig — (TIF) [file pcbi.1006877.s004.tif]

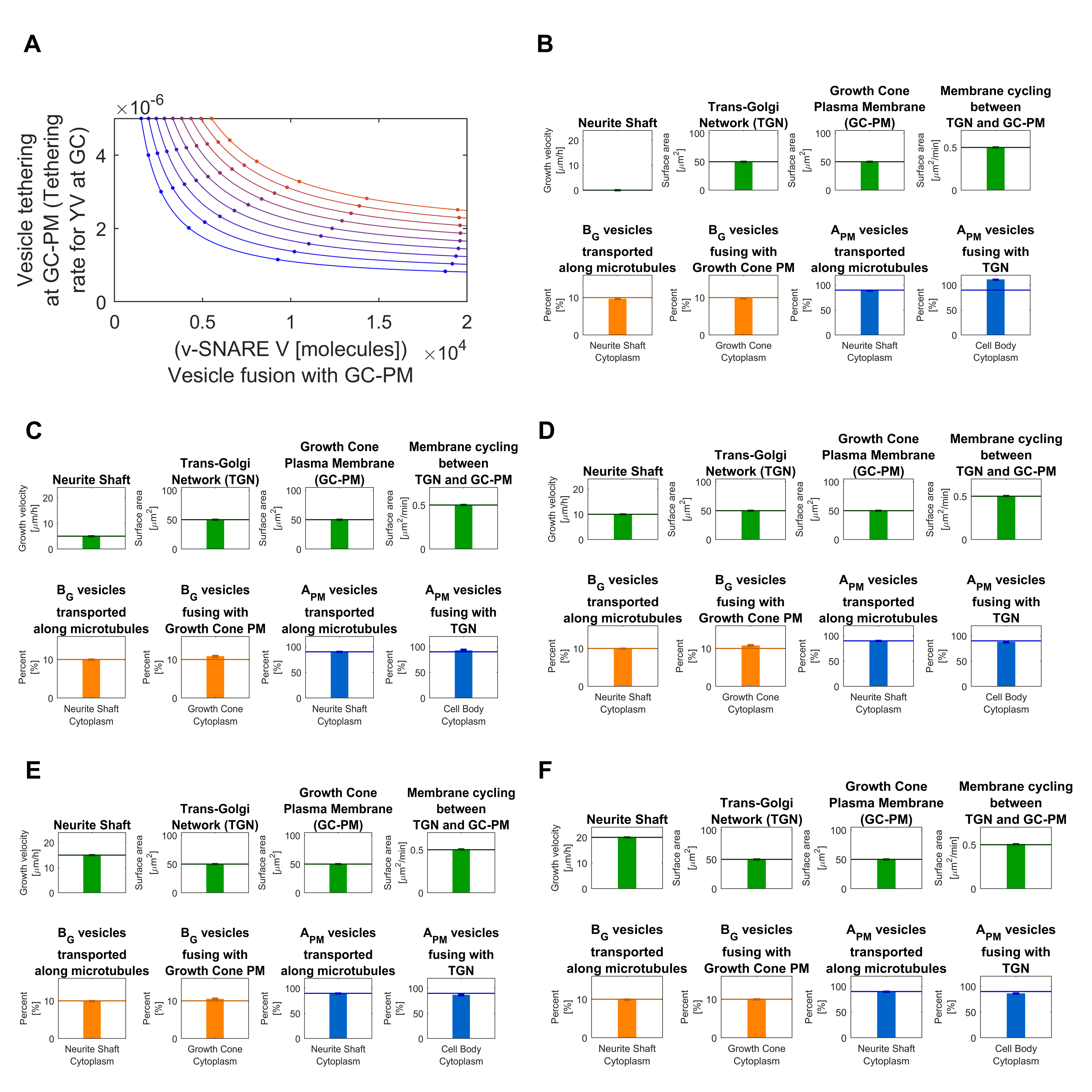

Supplement: S5 Fig — (A) To validate the predictions of kinetic parameters at steady state that allow NOG with a given velocity without violation of the model constraints, we selected 5 different combinations of v-SNARE V and tethering rates at the growth cone for each velocity (Dots in the diagram). (B-F) Results that were obtained for (B) no NOG and fixed NOG velocities of (C) 5 μm/h, (D) 10 μm/h, (E) 15 μm/h and (F) 20 μm/h. Similar results were obtained for the velocities 2.5 μm/h, 7.5 μm/h,12.5 μm/h and 17.5 μm/h (not shown). See Fig 4C for details. (TIF) [file pcbi.1006877.s005.tif]

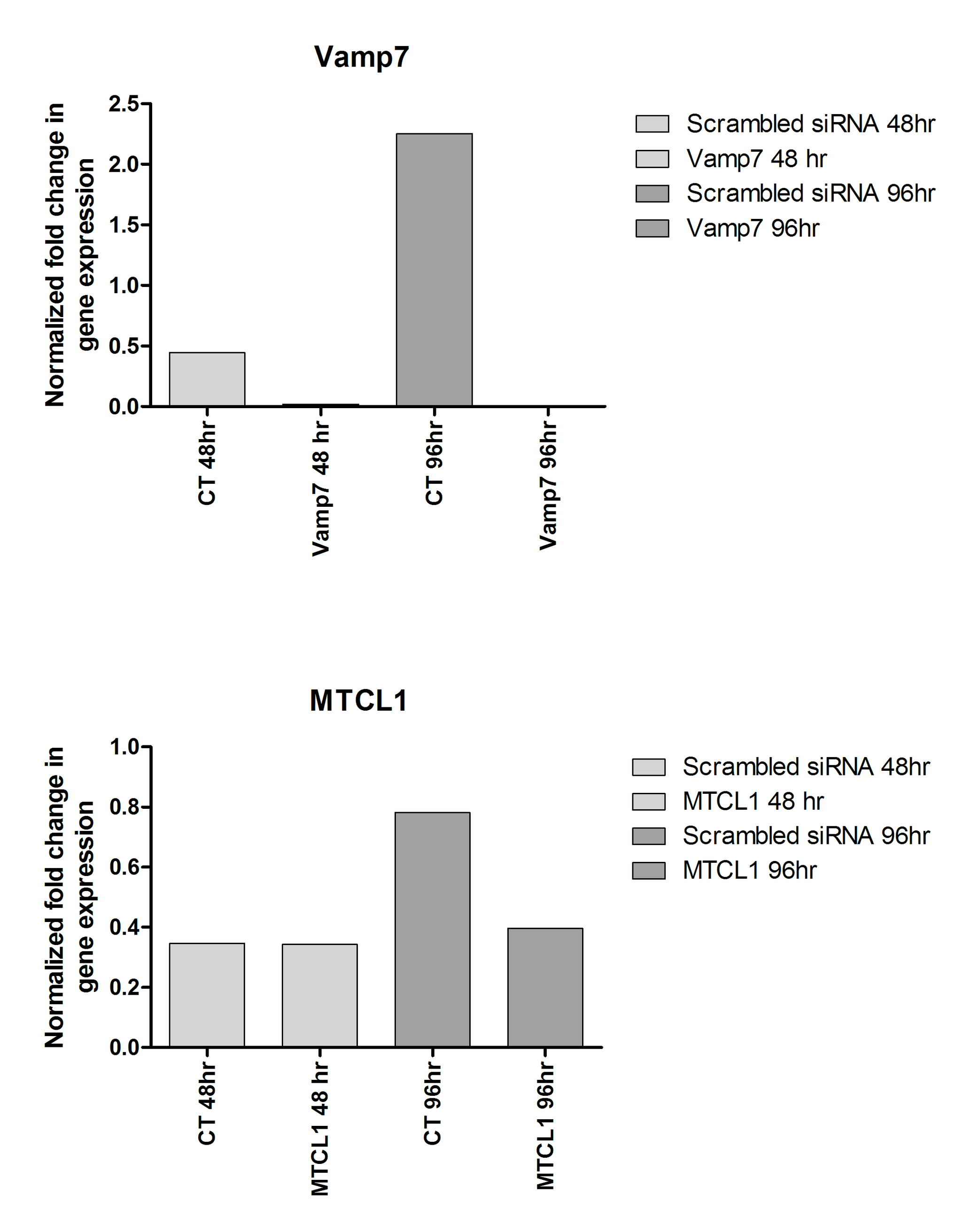

Supplement: S6 Fig — Primary neurons were transfected with siRNA against Vamp7, Mtcl1 or scrambled siRNAs. To document mRNA expression levels at the time of axotonomy, we harvested cells 48h after transfection and to document mRNA expression levels at the end of the outgrowth assay, we harvested cells 96h after transfection. Target mRNA expression levels were analyzed via RT-PCR. Results demonstrated a significant knock down of VAMP7 and a reduction by at least 50% of MTCL1 mRNA expression at both timepoints, using two different primer sets for both genes. (TIF) [file pcbi.1006877.s006.tif]
