# Supplementary material for: Dynamic balance between vesicle transport and microtubule growth enables neurite outgrowth
Source: PLoS Comput Biol. 2019 May 1;15(5):e1006877. doi: 10.1371/journal.pcbi.1006877 (PMC6546251; doi:10.1371/journal.pcbi.1006877)
Supplement: S1 Table — (DOCX) [file pcbi.1006877.s007.docx]

| **Reaction #** | **Description** | **Compartment** | **Reaction** | **Parameters** | **Molecules** |
| --- | --- | --- | --- | --- | --- |
| 1. | Membrane lipid production | TGN | $k_{\text{membrane production }}$ | $k_{\text{MP}}$ |  |
| 2. | Anterograde vesicle budding at Golgi | TGN🡺CBC | $f_{1}= w_{G}^{B}* S_{G}*{R_{1}}_{G}$ | $w_{G}^{B}$ | ${R_{1}}_{G}$ |
| 3. | Saturation function for carrying V-SNAREs to vesicles that bud from the TGN mediated by coat B | Anterograde  Vesicles | $\phi_{sv}^{B_{G}}=S^{\psi} \times\left( \frac{\frac{{sv}_{G}}{k_{sx}^{B}}}{1+ \frac{{sx}_{G}}{k_{sx}^{B}}+ \frac{{su}_{G}}{k_{su}^{B}}+ \frac{{sy}_{G}}{k_{sy}^{B}}+\frac{{sv}_{G}}{k_{sv}^{B}}} \right)$ | $k_{sx}^{B}$, $k_{su}^{B}$  $k_{sy}^{B}$, $k_{sv}^{B}$  $S^{\psi}$ | ${sx}_{G}, {su}_{G}$  ${sy}_{G}, {sv}_{G}$ |
| 4. | Saturation function for carrying ${kin}_{G}$ to vesicles that bud from the TGN mediated by coat B | Anterograde  Vesicles | $\phi_{kin}^{B_{G}}=M^{\psi}\times\left( \frac{\frac{{kin}_{G}}{k_{kin}^{B}}}{1+ \frac{{kin}_{G}}{k_{kin}^{B}}} \right)$ | $k_{kin}^{B}$  $M^{\psi}$ | ${kin}_{G}$ |
| 5. | Saturation function for carrying ${r_{1}}_{G}$ to vesicles that bud from the TGN mediated by coat B | Anterograde  Vesicles | $\phi_{r_{1}}^{A_{G}}=R^{\psi}\times\left( \frac{\frac{{r_{1}}_{G}}{k_{r_{1}}^{B}}}{1+ \frac{{r_{1}}_{G}}{k_{r_{1}}^{B}}} \right)$ | $k_{r_{1}}^{B}$  $R^{\psi}$ | ${r_{1}}_{G}$ |
| 6. | Kinesin mediated vesicle transport along microtubule in Cell body cytoplasm | CBC🡺NSC | ${(a) \#k}_{CBC}^{B_{G}}\text{=}\frac{{kin}_{CBC}^{B_{G}}\text{ }}{{\#N}_{CBC}^{B_{G}}},$  ${\left( b \right) fN}_{CBC}^{B_{G}}=1-\left( 1-f_{CBC}^{kin} \right)^{{\#k}_{CBC}^{B_{G}}},$  ${\left( c \right) f}_{2}=\frac{N_{CBC}^{B_{G}}}{T_{CBC}}*{fN}_{CBC}^{B_{G}}, T_{CBC}= \frac{L_{CBC}}{v_{k}}$ | $v_{k}, f_{CBC}^{kin}$,  ${\#k}_{CBC}^{B_{G}}$ | ${kin}_{CBC}^{B_{G}}$ |
| 7. | Kinesin mediated vesicle transport along microtubule in Neurite shaft cytoplasm | NSC 🡺 GCC | ${\left( a \right){\#k}_{NSC}^{B_{G}}\text{=}\frac{{kin}_{NSC}^{B_{G}}\text{ }}{{\#N}_{NSC}^{B_{G}}},}$  ${\left( b \right) fN}_{NSC}^{B_{G}}=1-\left( 1-f_{NSC}^{kin} \right)^{{\#k}_{NSC}^{B_{G}}},$  ${\left( c \right) f}_{3}=\frac{N_{NSC}^{B_{G}}}{T_{NSC}}*{fN}_{CBC}^{B_{G}}, T_{NSC}= \frac{L_{NSC}}{v_{k}}\text{ }$ | $v_{k}$, $f_{NSC}^{kin}$  ${\#k}_{NSC}^{B_{G}}$ | ${kin}_{NSC}^{B_{G}}$ |
| 8. | Vesicle fusion with target membrane, shown for example of anterograde vesicles with growth cone membrane | GCC🡺 GC | $f_{4}=\left( \begin{aligned} {\kappa_{XU}\times SX}_{GCC}^{B_{G}}\times{SU}_{PM} \\ + \kappa_{XU}\times{SU}_{GCC}^{B_{G}}\times{SX}_{PM} \\ + \kappa_{YV}\times{SY}_{GCC}^{B_{G}}\times{SV}_{PM} \\ + {\kappa_{YV}\times SV}_{GCC}^{B_{G}}\times{SY}_{PM} \end{aligned} \right)$*$\frac{\left( {1-fN}_{GCC}^{B_{G}} \right)\text{ }\text{*}S_{AVSA}}{R_{SCPVF}\text{ }}$ | $\kappa_{XU}$,$\kappa_{YV}$,  ${fN}_{GCC}^{B_{G}}$ | ${SX}_{GCC}^{B_{G}}$, ${SU}_{PM}$, ${SU}_{GCC}^{B_{G}}$, ${SX}_{PM}$, ${SY}_{GCC}^{B_{G}}$, ${SV}_{PM}$, ${SV}_{GCC}^{B_{G}}$, ${SY}_{PM}$ |
| 9. | Neurite shaft growth |  | $f_{5}$= Growth cone (GC) surface area – predefined GC area |  |  |
| 10. | Neurite shaft length |  | $L= s_{3}/(2 \times{\pi\times r}_{neurite})$ | $r_{neurite}$ |  |
