# Supplementary material for: Dynamic balance between vesicle transport and microtubule growth enables neurite outgrowth
Source: PLoS Comput Biol. 2019 May 1;15(5):e1006877. doi: 10.1371/journal.pcbi.1006877 (PMC6546251; doi:10.1371/journal.pcbi.1006877)
Supplement: S2 Table — Dissociation constants were selected to ensure preferred binding of proteins that are involved in anterograde transport to coat B and proteins that are involved in retrograde transport to coat A. High dissociation constants for stationary proteins for those coat proteins that would incorporate them into vesicles involved in back transport were selected to ensure that these proteins stay at their anticipated organelle. Orange: Proteins involved in anterograde movement, Blue: Proteins involved in retrograde movement, Italics: Proteins that cycle between the TGN and GC, Standard fonts: stationary molecules. kd values are taken from [19] which is normalized. (DOCX) [file pcbi.1006877.s008.docx]

| **Dissociation constants for interactions with Coat B** | |
| --- | --- |
| SNAREs | v-SNARE V ($k_{d}$=1), t-SNARE Y ($k_{d}$=1) < v-SNARE U ($k_{d}$=100) < t-SNARE X ($k_{d}$=10000) |
| Motor protein receptors | *kinesin receptor (*$k_{d}$*=0.1) < dynein receptor (*$k_{d}$*=10)* |
| Recruitment factors | Recruitment factor 1 ($k_{d}$=1) < recruitment factor 2 ($k_{d}$=100000) |
| **Dissociation constants for interactions with Coat A** | |
| SNAREs | v-SNARE U ($k_{d}$=1), t-SNARE X ($k_{d}$=1) < v-SNARE V ($k_{d}$=100) < t-SNARE Y ($k_{d}$=10000) |
| Motor protein receptors | *dynein receptor (*$k_{d}$*=0.1) < kinesin receptor (*$k_{d}$*=10)* |
| Recruitment factors | Recruitment factor 2 ($k_{d}$=1) < recruitment factor 1 ($k_{d}$=100000) |
