# Supplementary material for: Dynamic balance between vesicle transport and microtubule growth enables neurite outgrowth
Source: PLoS Comput Biol. 2019 May 1;15(5):e1006877. doi: 10.1371/journal.pcbi.1006877 (PMC6546251; doi:10.1371/journal.pcbi.1006877)
Supplement: S3 Table — A high fraction of MT-bound kinesin in the cell body cytoplasm ensures that vesicles are immediately transported forward after budding from the TGN. A low percentage of bound kinesin in the neurite shaft cytoplasm causes about 90% of the anterograde vesicles in the neurite shaft cytoplasm to be stationary and constitute a membrane mobilization reservoir that can be recruited upon demand. This fraction depends on the total amount of kinesin receptors (Fig 6B). No kinesin is bound to the MT in the GC cytoplasm, so that all anterograde vesicles are available for fusion with the GC membrane. The percentages of MT-bound dynein in the GC and CB cytoplasm were selected for the same reason. Since we do not assume that there is a membrane reservoir for retrograde vesicle, we selected a high percentage of bound dynein in the neurite shaft cytoplasm. (DOCX) [file pcbi.1006877.s009.docx]

| **Percentage of motor**  **Bound to microtubules** | **Cell body cytoplasm** | **Motor transport unit** | **Growth cone cytoplasm** |
| --- | --- | --- | --- |
| Percentage of kinesin bound/unbound | 95 /5 | 8 /92 | 0 /100 |
| Percentage of Dynein  bound/unbound | 0/100 | 84/ 16 | 95 /5 |
