# Supplementary material for: Dynamic balance between vesicle transport and microtubule growth enables neurite outgrowth
Source: PLoS Comput Biol. 2019 May 1;15(5):e1006877. doi: 10.1371/journal.pcbi.1006877 (PMC6546251; doi:10.1371/journal.pcbi.1006877)
Supplement: S4 Table — (DOCX) [file pcbi.1006877.s010.docx]

| **Reaction #** | **Description** | **Reaction** | **Parameters** | **Molecules** |
| --- | --- | --- | --- | --- |
| 11. | Nucleation of dynamic microtubules | $\frac{{d MT}_{Nu}}{dt}= \beta_{1}$ | $\beta_{1}$ |  |
| 12. | Average dynamic MT length | $L_{1}=-93.85+92.94x-752y-10.17x^{2}$  $-13.45xy+1159y^{2}+0.98x^{3}- 21.02x^{2}y +260.1xy^{2}-1588y^{3}$ | x, y | $Effective$  $tubulin,$ GTP hydrolysis |
| 13. | Degradation rate of dynamic microtubules | $\beta_{2}=0.152-0.086x+0.555y+0.142x^{2}$  $-0.152xy+0.286y^{2}-0.00065x^{3}+ 0.0072x^{2}y +0.0042xy^{2}-0.145y^{3}$ | x, y | $Effective$  $tubulin,$ GTP hydrolysis |
| 14. | Change in number of dynamic MTs | $\frac{{d N}_{1}}{dt}= \beta_{1}-\gamma\times N_{1}-\beta_{2}\times N_{1}$ | $\beta_{1}, \gamma, \beta_{2}$ |  |
| 15. | Length increase of stable MTs | $\frac{{d L}_{stbl}}{dt}= \gamma\times L_{dyn}$ | $\gamma,L_{dyn}$ |  |
| 16. | Combined length of dynamic MTs | $L_{dyn}= L_{1}\times N_{1}$ | $L_{1}, N_{1}$ |  |
| 17. | Microtubule bundle length | $L_{MTB}= \frac{L_{dyn}+L_{stbl}}{N_{2}}$ | $N_{2}$ |  |
