# Supplementary material for: Dynamic balance between vesicle transport and microtubule growth enables neurite outgrowth
Source: PLoS Comput Biol. 2019 May 1;15(5):e1006877. doi: 10.1371/journal.pcbi.1006877 (PMC6546251; doi:10.1371/journal.pcbi.1006877)
Supplement: S5 Table — (DOCX) [file pcbi.1006877.s011.docx]

| **Postulates governing SCP dynamics** | **Values** | **Reference** |
| --- | --- | --- |
| Trans Golgi Network size | 50 ${\mu m}^{2}$ | Assumed |
| Growth Cone size | 50 ${\mu m}^{2}$ | [37, 46] |
| Cycling membrane /Retrogradely transport membrane | 0.5 ${\mu m}^{2}$/min | [38] |
| Percentage of anterograde vesicles in NSC that are actively transported along microtubules | 10 % | [18] |
| Percentage of anterograde vesicles in GC cytoplasm that fuse with the growth cone | 10 % | [41] |
| Percentage of retrograde vesicles in NSC that are actively transported along microtubules | 90 % | Assumed |
| Percentage of retrograde vesicles in CB cytoplasm that fuse with the TGN | 90 % | Assumed |
| Length of dynamic microtubules | 20 $\mu m$ | [47] |
