# Supplementary material for: Dynamic balance between vesicle transport and microtubule growth enables neurite outgrowth
Source: PLoS Comput Biol. 2019 May 1;15(5):e1006877. doi: 10.1371/journal.pcbi.1006877 (PMC6546251; doi:10.1371/journal.pcbi.1006877)
Supplement: S6 Table — (DOCX) [file pcbi.1006877.s012.docx]

| **SL. No.** | **Symbols** | **Description** |
| --- | --- | --- |
| 1. | $S_{G}$ | Size of Golgi Compartment |
| 2. | $S_{PM}$ | Size of Growth Cone Plasma Membrane Compartment |
| 3. | $A_{G}$ | Coat A budded vesicles from Golgi |
| 4. | $B_{G}$ | Coat B budded vesicles from Golgi |
| 5. | $A_{PM}$ | Coat A budded vesicles from Growth Cone Plasma Membrane |
| 6. | $B_{PM}$ | Coat B budded vesicles from Growth Cone Plasma Membrane |
| 7. | $f_{1}$ | Forward rate of vesicles movement from Golgi to Cell Body Cytoplasm |
| 8. | $f_{2}$ | Forward rate of vesicles movement from Cell Body Cytoplasm to Neurite shaft cytoplasm |
| 9. | $f_{3}$ | Forward rate of vesicles movement from Neurite shaft cytoplasm to Growth Cone Cytoplasm |
| 10. | $f_{4}$ | Forward rate of vesicles movement from Growth Cone Cytoplasm to Growth Cone Plasma Membrane |
| 11. | $f_{5}$ | Forward rate of vesicles movement from Growth Cone Plasma Membrane to Growth Cone Cytoplasm |
| 12. | $f_{6}$ | Forward rate of vesicles movement from Growth Cone Cytoplasm to  Neurite shaft cytoplasm |
| 13. | $f_{7}$ | Forward rate of vesicles movement from Neurite shaft cytoplasm to  Cell Body Cytoplasm |
| 14. | $f_{8}$ | Forward rate of vesicles movement from Cell Body Cytoplasm to Golgi |
|  | $f_{9}$ | Neurite Shaft Growth rate |
| 15. | ${SX}_{G}$ | Total amount of SNARE X in Golgi compartment. |
| 16. | ${sx}_{G}$ | Concentration of SNARE X in Golgi compartment. Where ${sx}_{G}=\frac{{SX}_{G}}{S_{G}}$ |
| 17. | $R_{1_{G}}$ | Total amount of Recruitment factor 1 in Golgi compartment |
| 18. | $r_{1_{G}}$ | Concentration of Recruitment factor 1 in Golgi compartment. Where ${r_{1}}_{G}$ = ${R_{1}}_{G}$ /$S_{G}$ |
| 19. | ${Kin}_{G}$ | Total amount of Kinesin motor protein in Golgi compartment. |
| 20. | ${kin}_{G}$ | Concentration of Kinesin motor protein *Kin* in Golgi compartment. Where ${kin}_{G}=\frac{{Kin}_{G}}{S_{G}}$ |
| 21. | ${Dyn}_{G}$ | Total amount of Dynein motor protein in Golgi compartment. |
| 22. | ${dyn}_{G}$ | Concentration of Dynein motor protein *Dyn* in Golgi compartment. Where ${dyn}_{G}=\frac{{Dyn}_{G}}{S_{G}}$ |
| 23. | ${SX}_{GCC}^{B_{G}}$ | Number of SNAREs SX in the vesicles originated from Golgi with coat B in Growth Cone Cytoplasm (GCC) compartment |
| 24. | ${sx}_{GCC}^{B_{G}}$ | Average concentration of SNARE SX in the vesicles originated from Golgi with coat protein B in Growth Cone Cytoplasm (GCC) compartment. Where  ${sx}_{CBC}^{B_{G}}={SX}_{CBC}^{B_{G}}/ N_{\begin{aligned} CBC \\ \end{aligned}}^{B_{G}}$ |
| 25. | ${Kin}_{NSC}^{B_{G}}$ | Amount of Kinesin motor protein *Kin* in the vesicles originated from Golgi compartment with coat B in NSC compartment. |
| 26. | ${kin}_{NSC}^{B_{G}}$ | Be the average concentration of Kinesin *Kin* in vesicles originated in compartment G with coat protein B in NSC compartment. Where  ${kin}_{CBC}^{B_{G}}{= Kin}_{CBC}^{B_{G}}$ / $N_{CBC}^{B_{G}}$ |
| 27. | ${Dyn}_{NSC}^{A_{G}}$ | Amount of Dynein motor protein Dyn in the vesicles originated from Golgi compartment with coat A in NSC compartment. |
| 28. | ${dyn}_{NSC}^{A_{G}}$ | Average concentration of Dynein Dyn in vesicles originated in compartment G with coat protein A in NSC compartment. Where  ${dyn}_{CBC}^{A_{G}}=$ ${Dyn}_{CBC}^{A_{G}}$ / $N_{CBC}^{A_{G}}$ |
| 29. | ${R_{1}}_{CBC}^{B_{G}}$ | Total amount of recruitment factor 1 (*R_1_*) in the vesicles originated from Golgi compartment with coat B in CBC compartment. |
| 30. | ${r_{1}}_{CBC}^{B_{G}}$ | Average concentration recruitment factor 1 (*R_1_*) in the vesicles originated from Golgi compartment with coat B in CBC compartment. Where  ${{{r_{1}}_{CBC}^{B_{G}} =R}_{1}}_{CBC}^{B_{G}}$ / $N_{CBC}^{B_{G}}$ |
| 31. | ${R_{2}}_{CBC}^{B_{G}}$ | Total amount of recruitment factor 2 (*R_2_*) in the vesicles originated from Golgi compartment with coat B in CBC compartment. |
| 32. | ${r_{2}}_{CBC}^{B_{G}}$ | Average concentration recruitment factor 2 (*R_2_*) in the vesicles originated from Golgi compartment with coat B in CBC compartment. Where  ${{{r_{2}}_{CBC}^{B_{G}} =R}_{2}}_{CBC}^{B_{G}}$ / $N_{CBC}^{B_{G}}$ |
| 33. | $N_{CBC}^{B_{G}}$ | Number of vesicles originated from Trans Golgi Network with coat protein B in Cell Body Cytoplasm (CBC) compartment. |
| 34. | $k_{sx}^{B}$ | Dissociation constant of SNARE SX with coat protein B. |
| 35. | $k_{kin}^{A}$ | Dissociation constants of motor ${kin}_{G}$ from coat protein A |
| 36. | $k_{dyn}^{A}$ | Dissociation constants of motor ${dyn}_{G}$ from coat protein A |
| 37. | $k_{r_{1}}^{A}$ | Dissociation constants of recruitment factor 1 ($r_{1}$) from coat protein A |
| 38. | $k_{r_{2}}^{A}$ | Dissociation constants of recruitment factor 2 ($r_{2}$) from coat protein A |
| 39. | $\phi_{SV}^{B_{G}}$ | Denotes a saturation function for carrying SNARE *SV* to vesicles that  are initiated by coat B from Golgi |
| 40. | $\phi_{kin}^{B_{G}}$ | Denotes a saturation function for carrying motor protein *kin* to vesicles that  are initiated by coat B from Golgi |
| 41. | $\phi_{dyn}^{B_{G}}$ | Denotes a saturation function for carrying motor protein *dyn* to vesicles that  are initiated by coat B from Golgi |
| 42. | $\phi_{r_{1}}^{B_{G}}$ | Denotes a saturation function for carrying recruitment factor $r_{1}$ to vesicles that are initiated by coat B from Golgi |
| 43. | $\phi_{r_{2}}^{B_{G}}$ | Denotes a saturation function for carrying recruitment factor $r_{2}$ to vesicles that are initiated by coat B from Golgi |
| 44. | $w_{G}^{A}$ | Vesicles budding rate at Golgi with coat A |
| 45. | $w_{PM}^{A}$ | Vesicles budding rate at Growth Cone Plasma Membrane with coat A |
| 46. | $w_{G}^{B}$ | Vesicles budding rate at Trans Golgi Network with coat B |
| 47. | $w_{PM}^{B}$ | Vesicles budding rate at Growth Cone Plasma Membrane with coat B |
| 48. | $\kappa_{XU}$ | Fusion rate constant of vesicles (X, U) to target compartment |
| 49. | $\kappa_{YV}$ | Fusion rate constant of vesicles (Y, V) to target compartment |
| 50. | $v_{k}$ | Velocity of vesicle movement by one Kinesin receptor protein |
| 51. | $v_{d}$ | Velocity of vesicle movement by one Dynein receptor protein |
| 52. | $k_{MP}$ | Membrane lipid production rate |
| 53. | $S^{\psi}$ | SNARE binding spots per vesicle area |
| 54. | $M^{\psi}$ | Motor protein binding spots per vesicle area |
| 55. | $R^{\psi}$ | Cargo binding spots per vesicle area |
| 56. | $N_{1}$ | Number of dynamic microtubules |
| 57. | $N_{2}$ | Number of microtubules in neurite shaft cross section |
| 58. | $\beta_{1}$ | Nucleation rate of dynamic microtubule |
| 59. | $\beta_{2}$ | Degradation rate of dynamic microtubules |
| 60. | *a* | Scale parameter of power series method of degradation rate |
| 61. | *b* | Exponent parameter of power series method of degradation rate |
| 62. | $\theta_{1}$ | Shape Parameter (Gamma distribution) |
| 63. | $\theta_{2}$ | Scale parameter (Gamma distribution) |
| 64. | $L_{1}$ | Average dynamic microtubule length |
| 65. | γ | Rate of conversion from dynamic microtubules to stable microtubules |
| 66. | $L_{stbl}$ | Increase in length of Stable Microtubules |
| 67. | $L_{dyn}$ | Combined length of Dynamic Microtubules |
| 68. | $L_{MTB}$ | Microtubule scaffold length |
| 69. | $T_{CBC}$ | Average time taken by a vesicle to cross CBC compartment |
| 70. | $T_{NSC}$ | Average time taken by a vesicle to cross NSC compartment |
| 71. | $L_{CBC}$ | Microtubule scaffold length in CBC compartment |
| 72. | $L_{NSC}$ | Microtubule scaffold length in NSC compartment |
| 73. | ${\#k}_{CBC}^{B_{G}}$ | Number of kinesin motor receptor per vesicle in CBC compartment which is budded with Coat B from Golgi |
| 74. | ${\#k}_{NSC}^{B_{G}}$ | Number of kinesin motor receptor per vesicle in NSC compartment which is budded with Coat B from Golgi |
| 75. | $f_{CBC}^{kin}$ | Fraction of bound kinesin motor receptor in CBC compartment |
| 76. | $f_{NSC}^{kin}$ | Fraction of bound kinesin motor receptor in NSC compartment |
| 77. | ${fN}_{CBC}^{B_{G}}$ | Fraction of bound coat B budded vesicles in CBC compartment |
| 78. | ${fN}_{NSC}^{B_{G}}$ | Fraction of bound coat B budded vesicles in NSC compartment |
| 79. | ${fN}_{GCC}^{B_{G}}$ | Fraction of bound coat B budded vesicles in GCC compartment |
| 80. | $S_{AVSA}$ | Anterograde vesicle surface area |
| 81. | $R_{SCPVF}\text{ }$ | Required snare complex per vesicle fusion |

$\boldsymbol{Z}_{\boldsymbol{subscript}}^{\boldsymbol{Superscript}_{\boldsymbol{subscript of superscript}}}$

where,

*Superscript = Coat Protein*

*Subscript of superscript = Compartment*

*Subscript = Compartment*
