# Supplementary material for: Dynamic balance between vesicle transport and microtubule growth enables neurite outgrowth
Source: PLoS Comput Biol. 2019 May 1;15(5):e1006877. doi: 10.1371/journal.pcbi.1006877 (PMC6546251; doi:10.1371/journal.pcbi.1006877)
Supplement: S7 Table — The parameters set allows growth at 10 μm/h as shown in (Fig 4). (DOCX) [file pcbi.1006877.s013.docx]

| **Parameter** | **Value** | **Units** | **Reference** |
| --- | --- | --- | --- |
| Number of microtubules per cross-section | 20  (10 – 100) | # | [48] |
| Diameter of neurite | 1.0  (1.0 – 3.0) | µm | [49] |
| Length of growth cones | 10 | µm | [50] |
| Kinesin motor protein anterograde velocity | 1 | µm/s | [7, 8] |
| Dynein motor protein retrograde velocity | 0.65 | µm/s | [51, 52] |
| Vesicle diameter | 39.7 ± 6.6 | nm | [53] |
| Growth cone surface area | 70 - 200 | µm^2^ | [37] |
| Dissociation constant of SNARE SX with coat protein B.$(k_{sx}^{A} )$ | 1.00 | Molecules | [1] |
| Dissociation constant of SNARE SU with coat protein B.$(k_{su}^{A} )$ | 1.00 | Molecules | [1] |
| Dissociation constant of SNARE SX with coat protein B.$(k_{sx}^{B} )$ | 10000 | Molecules | [1] |
| Dissociation constant of SNARE SU with coat protein B.$(k_{su}^{B} )$ | 100 | Molecules | [1] |
| Dissociation constant of SNARE SY with coat protein B.$(k_{sy}^{A} )$ | 10000 | Molecules | [1] |
| Dissociation constant of SNARE SV with coat protein B.$(k_{sv}^{A} )$ | 100 | Molecules | [1] |
| Dissociation constant of SNARE SY with coat protein B.$(k_{sy}^{B} )$ | 1.00 | Molecules | [1] |
| Dissociation constant of SNARE SV with coat protein B.$(k_{sv}^{B} )$ | 1.00 | Molecules | [1] |
| Dissociation constants of recruitment factor 1 from coat protein A ($k_{r_{1}}^{A}$) | 1.00 | Molecules | Assumed |
| Dissociation constants of recruitment factor 1 from coat protein A ($k_{r_{1}}^{B}$) | 10000 | Molecules | Assumed |
| Dissociation constants of recruitment factor 2 from coat protein A ($k_{r_{2}}^{A}$) | 10000 | Molecules | Assumed |
| Dissociation constants of recruitment factor 2 from coat protein A ($k_{r_{2}}^{B}$) | 1.00 | Molecules | Assumed |
| Cargo binding spots per vesicle surface area | 2 | # |  |
| Dissociation constants of motor kinesin ${kin}_{G}$ from coat protein A ($k_{kin}^{A}$) | 10 | Molecules | Assumed |
| Dissociation constants of motor kinesin ${kin}_{G}$ from coat protein B ($k_{kin}^{B}$) | 0.1 | Molecules | Assumed |
| Dissociation constants of motor Dynein ${Dyn}_{G}$ from coat protein B ($k_{dyn}^{A}$) | 0.1 | Molecules | Assumed |
| Dissociation constants of motor Dynein ${Dyn}_{G}$ from coat protein B ($k_{dyn}^{B}$) | 10 | Molecules | Assumed |
| Length of CBC compartment | 1 | µm | Assumed |
| Length of GCC compartment | 2 | µm | Assumed |
| Length of NSC compartment | 20 | µm | Assumed |
| Scale parameter multiplier (a_1_) | 3.146e-12 |  | Predicted in pre-model |
| Scale parameter exponent (a_2_) | 1.0311 |  | Predicted in pre-model |
| Scale parameter multiplier (b_1_) | 8.7342e-19 |  | Predicted in pre -model |
| Scale parameter exponent (b_2_) | 4.2122 |  | Predicted in pre-model |
| Shape multiplier (m) | 0.02784 |  | Predicted in pre-model |
| Shape constant (b) | 0.15547 |  | Predicted in pre-model |
| Degradation multiplier (d1) | -12.78 |  | Predicted in pre-model |
| Degradation exponent (d2) | 2.93e10 |  | Predicted in pre-model |
| Anterograde vesicle surface area | 0.05 | µm^2^ | Predicted in pre-model |
| Retrograde vesicle surface area | 0.05 | µm^2^ | Predicted in pre-model |
| Snare binding spots per vesicle surface area | 1400 | # | Predicted |
| Motor binding spots per vesicle surface area | 140 | # | Predicted |
| SNARE Y production rate at TNG | 0.19772 | ${min}^{-1}$ | Predicted |
| SNARE V production rate at TNG | 8.337 | ${min}^{-1}$ | Predicted |
| SNARE X production rate at TNG | 0.91627 | ${min}^{-1}$ | Predicted |
| SNARE U production rate at TNG | 9.1851 | ${min}^{-1}$ | Predicted |
| Motor K production rate at TNG | 0.82659 | ${min}^{-1}$ | Predicted |
| Motor D production rate at TNG | 0.41034 | ${min}^{-1}$ | Predicted |
| Recruitment factor 1 production rate at TNG | 1.3339e-06 | ${min}^{-1}$ | Predicted |
| Recruitment factor 2 production rate at TNG | 6.5315e-07 | ${min}^{-1}$ | Predicted |
| Vesicles budding rate at TGN with coat A  ( $w_{G}^{A}$) | 1.0024e-05 | µm^2^ | Predicted |
| Vesicles budding rate at Plasma Membrane with coat A ( $w_{PM}^{A}$) | 0.00010047 | µm^2^ | Predicted |
| Vesicles budding rate at TGN with coat B  ( $w_{G}^{B}$) | 0.00021089 | µm^2^ | Predicted |
| Vesicles budding rate at Plasma Membrane with coat A ( $w_{PM}^{B}$) | 2.1089e-05 | µm^2^ | Predicted |
| Tethering rate constant of vesicles (X, U) to target compartment Golgi (${\kappa_{XU}}_{G}$) | 8e-06 | 1/ (molecules min) | Predicted |
| Tethering rate constant of vesicles (X, U) to target compartment Growth Cone Plasma Membrane (${\kappa_{XU}}_{PM}$) | 8e-07 | 1/ (molecules min) | Predicted |
| Tethering rate constant of vesicles (Y, V) to target compartment Golgi (${\kappa_{YV}}_{G}$) | 2e-07 | 1/ (molecules min) | Predicted |
| Tethering rate constant of vesicles (Y, V) to target compartment Growth Cone Plasma Membrane (${\kappa_{YV}}_{PM}$) | 2e-06 | 1/ (molecules min) | Predicted |
| Required snare complex per vesicle fusion ($R_{SCPVF}\text{ )}$ | 5.00 | # | Predicted |
| Fraction of bound kinesin motor with microtubules in CBC compartment | 95 | percent | Assumed |
| Fraction of bound kinesin motor with microtubules in NSC compartment | 8.15 | Percent | Assumed |
| Fraction of bound kinesin motor with microtubules in GCC compartment | 0 | Percent | Assumed |
| Fraction of bound dynein motor with microtubules in CBC compartment | 0 | Percent | Assumed |
| Fraction of bound dynein motor with microtubules in NSC compartment | 84 | Percent | Assumed |
| Fraction of bound dynein motor with microtubules in CBC compartment | 95 | percent | Assumed |
| Membrane production rate at TGN | 0.55447 | µm^2^ | Predicted |
| Nucleation rate of microtubules | 2.7929 | 1/min | Predicted |
| Stabilization rate of dynamic microtubules | 0.0083 | 1/min | Predicted |
|  |  |  |  |
|  |  |  |  |
| **Initial Values** |  |  | Predicted |
| Trans Golgi Network (TGN) | 50 | µm^2^ | Predicted |
| Coat B budded vesicles membrane surface area from TGN in CBC | 0.03 | µm^2^ | Predicted |
| Coat B budded vesicles membrane surface area from TGN in NSC | 3.4199 | µm^2^ | Predicted |
| Coat B budded vesicles membrane surface area from TGN in GCC | 10.2597 | µm^2^ | Predicted |
| Growth cone plasma membrane | 50 | µm^2^ | Predicted |
| Coat A budded vesicles membrane surface area from TGN in CBC | 2.2911e-06 | µm^2^ | Predicted |
| Coat A budded vesicles membrane surface area from TGN in NSC | 0.0008038 | µm^2^  µm^2^ | Predicted |
| Coat A budded vesicles membrane surface area from TGN in GCC | 0.0067794 |  | Predicted |
| Coat B budded vesicles membrane surface area from plasma membrane in CBC | 0.01252 | µm^2^ | Predicted |
| Coat B budded vesicles membrane surface area from plasma membrane in NSC | 6.5014e-05 | µm^2^ | Predicted |
| Coat B budded vesicles membrane surface area from plasma membrane in GCC | 4.0126e-05 | µm^2^ | Predicted |
| Coat A budded vesicles membrane surface area from plasma membrane in CBC | 0.55556 | µm^2^ | Predicted |
| Coat A budded vesicles membrane surface area from plasma membrane in NSC | 0.2849 | µm^2^ | Predicted |
| Coat A budded vesicles membrane surface area from plasma membrane in GCC | 0.14 | µm^2^ | Predicted |
| Neurite shaft surface area | 62.8319 | µm^2^ | Predicted |
| SNAREs Y in TGN compartment | 11.6606 | molecules | Predicted |
| Total vesicle snare Y with Coat B budded from TNG in CBC | 0.17787 | molecules | Predicted |
| Total vesicle snare Y with Coat B budded from TNG in NSC | 20.2772 | Molecules | Predicted |
| Total vesicle snare Y with Coat B budded from TNG in GCC | 60.8315 | Molecules | Predicted |
| SNAREs Y in GC compartment | 20000 | Molecules | Predicted |
| Total vesicle snare Y with Coat A budded from TNG in CBC | 1.9228e-11 | Molecules | Predicted |
| Total vesicle snare Y with Coat A budded from TNG in NSC | 6.746e-09 | Molecules | Predicted |
| Total vesicle snare Y with Coat A budded from TNG in GCC | 0.00011986 | Molecules | Predicted |
| Total vesicle snare Y with Coat B budded from GC in CBC | 10.449 | Molecules | Predicted |
| Total vesicle snare Y with Coat B budded from GC in NSC | 0.054339 | Molecules | Predicted |
| Total vesicle snare Y with Coat B budded from GC in GCC | 0.033537 | Molecules | Predicted |
| Total vesicle snare Y with Coat A budded from GC in CBC | 6.7271 | Molecules | Predicted |
| Total vesicle snare Y with Coat A budded from GC in NSC | 3.4498 | Molecules | Predicted |
| Total vesicle snare Y with Coat A budded from GC in GCC | 1.6952 | Molecules | Predicted |
| SNAREs V in TGN compartment | 9.8607 | Molecules | Predicted |
| Total vesicle snare V with Coat B budded from TNG in CBC | 7.5 | Molecules | Predicted |
| Total vesicle snare V with Coat B budded from TNG in NSC | 854.9775 | Molecules | Predicted |
| Total vesicle snare V with Coat B budded from TNG in GCC | 2564.9324 | Molecules | Predicted |
| SNAREs V in GC compartment | 8432.9044 | Molecules | Predicted |
| Total vesicle snare V with Coat A budded from TNG in CBC | 1.2923e-07 | Molecules | Predicted |
| Total vesicle snare V with Coat A budded from TNG in NSC | 4.5337e-05 | molecules | Predicted |
| Total vesicle snare V with Coat A budded from TNG in GCC | 0.00049928 | Molecules | Predicted |
| Total vesicle snare V with Coat B budded from GC in CBC | 7.0517 | Molecules | Predicted |
| Total vesicle snare V with Coat B budded from GC in NSC | 0.036543 | Molecules | Predicted |
| Total vesicle snare V with Coat B budded from GC in GCC | 0.022554 | Molecules | Predicted |
| Total vesicle snare V with Coat A budded from GC in CBC | 283.6456 | Molecules | Predicted |
| Total vesicle snare V with Coat A budded from GC in NSC | 145.4593 | Molecules | Predicted |
| Total vesicle snare V with Coat A budded from GC in GCC | 71.4787 | Molecules | Predicted |
| SNAREs X in TGN compartment | 19999.0837 | Molecules | Predicted |
| Total vesicle snare X with Coat B budded from TNG in CBC | 0.82428 | Molecules | Predicted |
| Total vesicle snare X with Coat B budded from TNG in NSC | 93.9654 | Molecules | Predicted |
| Total vesicle snare X with Coat B budded from TNG in GCC | 281.8961 | Molecules | Predicted |
| SNARE X in GC compartment | 2 | Molecules | Predicted |
| Total vesicle snare X with Coat A budded from TNG in CBC | 0.0019236 | Molecules | Predicted |
| Total vesicle snare X with Coat A budded from TNG in NSC | 0.67487 | Molecules | Predicted |
| Total vesicle snare X with Coat A budded from TNG in GCC | 5.6971 | Molecules | Predicted |
| Total vesicle snare X with Coat B budded from GC in CBC | 0.00048599 | molecules | Predicted |
| Total vesicle snare X with Coat B budded from GC in NSC | 5.4461e-10 | Molecules | Predicted |
| Total vesicle snare X with Coat B budded from GC in GCC | 3.3613e-10 | Molecules | Predicted |
| Total vesicle snare X with Coat A budded from GC in CBC | 31.1738 | Molecules | Predicted |
| Total vesicle snare X with Coat A budded from GC in NSC | 15.9865 | Molecules | Predicted |
| Total vesicle snare X with Coat A budded from GC in GCC | 7.8558 | Molecules | Predicted |
| SNAREs U in TGN compartment | 1995.7062 | Molecules | Predicted |
| Total vesicle snare U with Coat B budded from TNG in CBC | 8.263 | Molecules | Predicted |
| Total vesicle snare U with Coat B budded from TNG in NSC | 941.9516 | Molecules | Predicted |
| Total vesicle snare U with Coat B budded from TNG in GCC | 2825.8547 | Molecules | Predicted |
| SNARE U in GC compartment | 92.9076 | Molecules | Predicted |
| Total vesicle snare U with Coat A budded from TNG in CBC | 0.001279 | Molecules | Predicted |
| Total vesicle snare U with Coat A budded from TNG in NSC | 0.44874 | Molecules | Predicted |
| Total vesicle snare U with Coat A budded from TNG in GCC | 3.7795 | Molecules | Predicted |
| Total vesicle snare U with Coat B budded from GC in CBC | 0.0011828 | Molecules | Predicted |
| Total vesicle snare U with Coat B budded from GC in NSC | 3.6171e-06 | Molecules | Predicted |
| Total vesicle snare U with Coat B budded from GC in GCC | 2.2324e-06 | Molecules | Predicted |
| Total vesicle snare U with Coat A budded from GC in CBC | 312.5 | Molecules | Predicted |
| Total vesicle snare U with Coat A budded from GC in NSC | 160.2564 | Molecules | Predicted |
| Total vesicle snare U with Coat A budded from GC in GCC | 78.75 | Molecules | Predicted |
| Kinesin receptors K in TGN compartment | 1.0757 | Molecules | Predicted |
| Total kinesin receptors K with Coat B budded from TNG in CBC | 0.7436 | Molecules | Predicted |
| Total kinesin receptors K with Coat B budded from TNG in NSC | 84.7687 | Molecules | Predicted |
| Total kinesin receptors K with Coat B budded from TNG in GCC | 254.306 | Molecules | Predicted |
| kinesin receptors K in GC compartment | 283.1802 | Molecules | Predicted |
| Total kinesin receptors K with Coat A budded from TNG in CBC | 7.0251e-07 | Molecules | Predicted |
| Total kinesin receptors K with Coat A budded from TNG in NSC | 0.00024645 | Molecules | Predicted |
| Total kinesin receptors K with Coat A budded from TNG in GCC | 0.0020644 | Molecules | Predicted |
| Total kinesin receptors K with Coat B budded from GC in CBC | 1.6756 | Molecules | Predicted |
| Total kinesin receptors K with Coat B budded from GC in NSC | 0.0087054 | Molecules | Predicted |
| Total kinesin receptors K with Coat B budded from GC in GCC | 0.0053728 | Molecules | Predicted |
| Total kinesin receptors K with Coat A budded from GC in CBC | 28.1227 | Molecules | Predicted |
| Total kinesin receptors K with Coat A budded from GC in NSC | 14.4219 | Molecules | Predicted |
| Total kinesin receptors K with Coat A budded from GC in GCC | 7.0869 | Molecules | Predicted |
| Dynein receptor D in TGN compartment | 48.1803 | Molecules | Predicted |
| Total dynein receptor D with Coat B budded from TNG in CBC | 0.36914 | Molecules | Predicted |
| Total dynein receptor D with Coat B budded from TNG in NSC | 42.0812 | Molecules | Predicted |
| Total dynein receptor D with Coat B budded from TNG in GCC | 126.2437 | Molecules | Predicted |
| Dynein receptor D in GC compartment | 1.0938 | Molecules | Predicted |
| Total dynein receptor D with Coat A budded from TNG in CBC | 0.00030677 | Molecules | Predicted |
| Total dynein receptor D with Coat A budded from TNG in NSC | 0.10763 | Molecules | Predicted |
| Total dynein receptor D with Coat A budded from TNG in GCC | 0.90727 | Molecules | Predicted |
| Total dynein receptor D with Coat B budded from GC in CBC | 0.0038582 | Molecules | Predicted |
| Total dynein receptor D with Coat B budded from GC in NSC | 1.9925e-05 | Molecules | Predicted |
| Total dynein receptor D with Coat B budded from GC in GCC | 1.2297e-05 | Molecules | Predicted |
| Total dynein receptor D with Coat A budded from GC in CBC | 13.9608 | Molecules | Predicted |
| Total dynein receptor D with Coat A budded from GC in NSC | 7.1594 | Molecules | Predicted |
| Total dynein receptor D with Coat A budded from GC in GCC | 3.5181 | Molecules | Predicted |
| Recruitment factor 1 in TGN compartment | 100 | Molecules | Predicted |
| Total recruitment factor 1 with Coat B budded from TNG in CBC | 1.2e-06 | Molecules | Predicted |
| Total recruitment factor 1 with Coat B budded from TNG in NSC | 0.00013679 | Molecules | Predicted |
| Total recruitment factor 1 with Coat B budded from TNG in GCC | 0.00041038 | Molecules | Predicted |
| recruitment factor 1 in GC compartment | 0.0020423 | Molecules | Predicted |
| Total recruitment factor 1 with Coat A budded from TNG in CBC | 3.0544e-05 | Molecules | Predicted |
| Total recruitment factor 1 with Coat A budded from TNG in NSC | 0.010716 | Molecules | Predicted |
| Total recruitment factor 1 with Coat A budded from TNG in GCC | 0.090358 | Molecules | Predicted |
| Total recruitment factor 1 with Coat B budded from GC in CBC | 7.4581e-11 | Molecules | Predicted |
| Total recruitment factor 1 with Coat B budded from GC in NSC | 3.6518e-13 | Molecules | Predicted |
| Total recruitment factor 1 with Coat B budded from GC in GCC | 2.2539e-13 | Molecules | Predicted |
| Total recruitment factor 1 with Coat A budded from GC in CBC | 4.5382e-05 | Molecules | Predicted |
| Total recruitment factor 1 with Coat A budded from GC in NSC | 2.3273e-05 | Molecules | Predicted |
| Total recruitment factor 1 with Coat A budded from GC in GCC | 1.1436e-05 | Molecules | Predicted |
| Recruitment factor 2 in TGN compartment | 0.00048965 | Molecules | Predicted |
| Total recruitment factor 2 with Coat B budded from TNG in CBC | 5.8758e-07 | Molecules | Predicted |
| Total recruitment factor 2 with Coat B budded from TNG in NSC | 6.69824e-05 | Molecules | Predicted |
| Total recruitment factor 2 with Coat B budded from TNG in GCC | 0.00020095 | Molecules | Predicted |
| Recruitment factor 2 in GC compartment | 100 | Molecules | Predicted |
| Total recruitment factor 2 with Coat A budded from TNG in CBC | 1.1325e-14 | Molecules | Predicted |
| Total recruitment factor 2 with Coat A budded from TNG in NSC | 3.9661e-12 | Molecules | Predicted |
| Total recruitment factor 2 with Coat A budded from TNG in GCC | 3.829e-11 | Molecules | Predicted |
| Total recruitment factor 2 with Coat B budded from GC in CBC | 0.16687 | Molecules | Predicted |
| Total recruitment factor 2 with Coat B budded from GC in NSC | 0.00086636 | Molecules | Predicted |
| Total recruitment factor 2 with Coat B budded from GC in GCC | 0.0005347 | Molecules | Predicted |
| Total recruitment factor 2 with Coat A budded from GC in CBC | 2.2222e-05 | Molecules | Predicted |
| Total recruitment factor 2 with Coat A budded from GC in NSC | 1.1396e-05 | Molecules | Predicted |
| Total recruitment factor 2 with Coat A budded from GC in GCC | 5.5999e-06 | Molecules | Predicted |
| Effective tubulin | 9 | µM | Assumed |
