# Supplementary material for: Dynamic balance between vesicle transport and microtubule growth enables neurite outgrowth
Source: PLoS Comput Biol. 2019 May 1;15(5):e1006877. doi: 10.1371/journal.pcbi.1006877 (PMC6546251; doi:10.1371/journal.pcbi.1006877)
Supplement: S8 Table — (DOCX) [file pcbi.1006877.s014.docx]

| REAGENT or RESOURCE | SOURCE | IDENTIFIER |
| --- | --- | --- |
| Antibodies | | |
| Anti- tubulin β –III (TUJ1 clone) made in mouse | BioLegend | 801201 |
| Mountant PermaFluor | ThermoFisher Sci | TA-030-FM |
| Goat anti-Mouse IgG Secondary- Alexa Fluor488 | ThermoFisher Sci | A-11001 |
| Neurobasal-A Medium | ThermoFisher Sci | 10888022 |
| B-27 Supplement (50X), serum free | ThermoFisher Sci | 17504044 |
| L-Glutamine (200mM) | ThermoFisher Sci | 25030081 |
| Penicillin-Streptomycin (5,000 U/ml) | ThermoFisher Sci | 15070063 |
| OptiPrep Density Gradient Medium | Sigma-Aldrich | D1556 |
| Papain, aseptically filled | Sigma-Aldrich | P5306 |
| Deoxyribonuclease I from bovine pancreas | Sigma-Aldrich | DN25 |
| Sterile Cell Strainers (40µm) | Fisher Scientific | 22-363-547 |
| Accell siRNA delivery media | Dharmacon | B00500-100 |
| 5x siRNA buffer | Dharmacon | B002000-UB-100 |
| Recombinant DNA | | |
| Mtcl1 SiRNA1 GUGUUUUAUUUCAUGCUUC | Dharmacon | E-085290-00 |
| Mtcl1 SiRNA2 UCAGAGUGUUCUAAGUUAA | Dharmacon | E-085290-00 |
| Mtcl1 SiRNA3 CCCUGAAGCAGAACAUUUU | Dharmacon | E-085290-00 |
| Mtcl1 SiRNA4 GCUUUGUCCCUGGAUGAUG | Dharmacon | E-085290-00 |
| Vamp7 SiRNA1 CCAUCAUCGUCGUUGUAUC | Dharmacon | E-094480-00 |
| Vamp7 SiRNA2 GGAUUGUGUAUCUUUGCAU | Dharmacon | E-094480-00 |
| Vamp7 SiRNA3 UCAUGGCAAUUAUUUGUUU | Dharmacon | E-094480-00 |
| Vamp7 SiRNA4 CCAUGAACAGUGAGUUUUC | Dharmacon | E-094480-00 |
| Accell non-targeting pool | Dharmacon | D-001910-10-20 |
| Software and Algorithms | | |
| ImageJ | ImageJ | ImageJ 1.49v |
| GraphPad Prism | GraphPad Software | GraphPad Prism7 |
| Adobe Photoshop CS5 | Adobe | Photoshop CS5 |
| MATLAB | MATHWORKS | R2017b |
| MetaMorph® | Neurite outgrowth module | MetaMorph 7.7 |
| Other | | |
| Xona Microfluidic chambers | Xona Microfluidics | SND150 |
| 50mm Glass Bottom Dishes No. 1.5 | MatTek Corp. | P50G-1.5-30-F |
| LSM 880 Laser Scanning Microscope with Airyscan | Zeiss Microscopy | Zeiss LSM 880 |
