# Supplementary material for: Dynamic balance between vesicle transport and microtubule growth enables neurite outgrowth
Source: PLoS Comput Biol. 2019 May 1;15(5):e1006877. doi: 10.1371/journal.pcbi.1006877 (PMC6546251; doi:10.1371/journal.pcbi.1006877)
Supplement: S1 Text — (DOCX) [file pcbi.1006877.s015.docx]

**Supporting Information:**

**Dynamic model of vesicle transport and microtubule growth**

**Base vesicle transport model**

Our vesicle transport model of membrane delivery to the growing neurite tip is based on a dynamical model of bidirectional membrane lipid and transmembrane protein transport between two organelles [1]. In this model vesicle budding is mediated by two different coat proteins, coat A and coat B, and vesicle fusion by two different sets of complementary SNAREs, SNAREs Y and V and SNAREs X and U. Two different recruitment factors favor the budding of each of the two different coat proteins. Coat A has a high affinity towards SNAREs X and U, coat B towards SNAREs Y and V. Consequently, vesicles that bud with coat A have more SNAREs X and U and preferentially fuse with that organelle that has more X and U SNAREs. After fusion with the organelle all SNAREs of the vesicle are transmitted to this organelle, further increasing the SNARE set XU content of this organelle. The same accounts for SNARE set 2 and coat B. The inclusion of recruitment factors that recruit coat proteins to an organelle and initiate vesicle budding, secure that each coat protein specifically buds from one organelle but not the other, fulfilling a basic principle of intracellular vesicle transport. Recruitment factor 1 recruits coat protein B to the organelle membrane and is transported by vesicles that bud with coat protein A. The same accounts for recruitment factor 2 and coat protein B. At steady state the model resembles the intracellular transport between two organelles. Recruitment factor 1 recruits coat B to the donor organelle, coat B mediates the budding of anterograde moving vesicles that fuse with the target organelle via complex formation between SNAREs Y and V. Similarly, retrograde transport is mediated by the interplay of recruitment factor 2, coat A and SNAREs X and U.

The model does not track individual vesicles but assumes that all vesicles of one type (i.e. those vesicles that bud from the same donor membrane with the same coat protein) form a combined compartment in the cytoplasm between the organelles. Biologically, this assumes that there is rapid and un-limited diffusion of all vesicle proteins between all vesicles of the same type. Once a vesicle buds from an organelle the concentration of all its protein components (i.e. SNAREs and recruitment factors) immediately equilibrates with the concentration of the protein components of all other vesicles of the same type. This assumption is reasonable, since at the steady-state there should be no differences between the protein content of a newly budded vesicle and previously budded vesicles. The assumption is also reasonable for our purpose, since we want to model the steady-state that enable continuous neurite outgrowth with a predefined outgrowth velocity.

**Inclusion of microtubule-based vesicle transport**

During neurite outgrowth new membrane is synthesized in the cell body [2, 3] and transported to the neurite tip via microtubule-based vesicle transport where it is inserted into the plasma membrane of the growth cone [3, 4, 5]. Anterograde moving vesicles bud from the TGN and are loaded on the microtubules [6], travel along the axonal shaft via kinesin mediated active transport [7, 8] and fuse with the growth cone plasma membrane (GC-PM), thereby delivering new membrane to the growing neurite [4]. Directed retrograde vesicle movement from the growth cone membrane back to the TGN is a similar process that is mediated by the motor protein dynein. To account for microtubule-based vesicle movements, we replaced the cytoplasm of the original model by three new vesicle compartments, the cell body cytoplasm (CBC), the neurite shaft cytoplasm (NSC) and the Growth cone cytoplasm (GCC) and introduced two new transmembrane proteins, a kinesin and a dynein receptor. Each vesicle compartment contains microtubules that bind kinesin and dynein attached vesicles. The compartments differ in the affinity of MTs for kinesin and dynein. We model the different affinities by different fractions of MT bound kinesin molecules, the higher the affinity, the higher the fraction bound kinesin. The cell body cytoplasm has a very high affinity of the MTs for kinesin, resembling the observation that anterograde vesicles are loaded to the microtubule in a MACF dependent process right after budding and assuring immediate anterograde vesicle movement [6]. Kinesin competes with the microtubule associated protein tau for microtubule binding spots [9, 10, 11, 12, 13, 14]. Multiple studies report an increasing gradient of tau from the base of the neurite to the growth cone [15, 16, 17]. We included this gradient of tau mediated inhibition of kinesin binding in our model by decreasing fraction of bound kinesin in the neurite shaft cytoplasm and further in the growth cone cytoplasm. Such a decrease is also in agreement with the experimental observation that only a few vesicles (~9%) in axons are moving forward, while the majority of vesicles only shows random diffusion-based movements [18], implicating that the neurite shaft cytoplasm compartment functions as a vesicle reservoir for on-demand membrane recruitment at the growth cone. Concordantly, neurites continue growing for about 6h after disruption of the TGN, the source of newly generated membrane [19]. The lower affinity for kinesin for microtubules in the growth cone cytoplasm ensures an almost complete dissociation of vesicles from the microtubule so that they are available for fusion with the growth cone membrane. The binding of dynein to the microtubules in the growth cone is not significantly affected by tau [9], allowing for a higher affinity. In contrast to kinesin, affinity to the MTs for dynein is also high in the NSC compartment, assuming that there is no reservoir for backward moving vesicles and that these vesicles are transported back to the TGN without any hesitation as it was observed for components of the degradative pathways such as autophagosomes [20, 21]. In the cell body cytoplasm, the fraction of MT bound dynein is very low as for kinesin in the growth cone cytoplasm, allowing the fusion of retrograde vesicles with the TGN.

To allow the binding of vesicles to the microtubule through the motor proteins, we introduce two new transmembrane proteins, the dynein and the kinesin receptors (or adaptor) [22]. Although it was reported that vesicle SNAREs can also interact with kinesin [6], we do not consider such an interaction in our model at this stage. Our model does not explicitly contain dynein or kinesin molecules, but we assume that both motor proteins exist under saturating conditions and motor protein receptors are always bound to dynein or kinesin. Another assumption that we made is that kinesin receptors only on anterograde, but not on retrograde vesicles, are bound to active kinesin, while only dynein receptors on retrograde, but not on anterograde vesicles, are bound to active dynein. The reason for this assumption is the need for a back transport of the relevant receptor to the corresponding organelle, i.e. kinesin receptor to the TGN and dynein receptor to the growth cone membrane. In agreement with our assumption, experimental data show that backward moving vesicles contain kinesin as a passive passenger [20, 21, 23] and kinesin positively regulates dynein mediated retrograde transport of prion protein vesicles [24]. Dynein is transported to the growth cone through direct interaction with kinesin [25] suggesting it’s binding to the dynein receptor at the growth cone and its dissociation from the receptor before fusion with the TGN. The preferred binding of the kinesin receptor to coat B and of the dynein receptor to coat A is modeled in the same approach as the binding of the recruitment factors to each coat proteins.

**Site specific budding machinery**

Endocytosis is mediated by specialized mechanism such as clathrin- or caveolin-mediated endocytosis [16, 27, 28], while different mechanisms are involved in the budding of vesicles from the TGN [29, 30]. Budding mechanisms from various organelles are initiated by membrane factors that recruit the coat proteins to the membrane of the donor organelle. Such membrane factors are implemented in the original model where they are labeled as cargo [1]. Regulatory and structural coat components consist of proteins that are either in the cytoplasm or bind the donor membrane as peripheral membrane proteins. In both cases they are localized at their preferred site of action, e.g. components that mediate caveolin-mediated endocytosis are next to the plasma membrane and not to the TGN. We incorporate such locally restricted availability of the coat components by the introduction of site-specific budding rate constants for the two coat proteins A and B in our model.

**Recruitment of transmembrane proteins into the budding vesicles**

To calculate the recruitment of SNAREs, recruitment factors and motor protein receptors into the budding vesicles, we used the same formulas that were described in the original model.

**Movement of budded vesicles**

In contrast to the original model we do not allow back fusion of newly budded vesicles to the donor organelle but assume that they are immediately moved into the anticipated direction. Regulatory mechanisms that prevent the back fusion have been identified for ER budded vesicles [31]. Endocytosed vesicles are transported via an actin comet tail into the cell interior [32], a mechanism that should significantly lower the chance of back fusion of these vesicles to the plasma membrane. We assume that the F-actin network of the lamellipodia in the growth cone also triggers such an immediate movement of endocytosed vesicles into the central domain of the growth cone, where they can interact with the microtubule allowing their active dynein mediated back transport to the cell body. Simultaneously, a mechanism that mediates loading of TGN-derived vesicles onto the microtubules during budding has been described in neurons [6], that should allow the immediate removal of these vesicles from the TGN.

**Calculation of vesicle transport rates along the microtubule**

Vesicles and organelles travel along the microtubule in the axon with a velocity between 0.5-3.5 µm/min [4, 6, 33], not much faster than the speed by which single kinesin molecules move along the microtubule [7, 8]. Since the size of the kinesin attached cargo does not seem to influence the transport speed, we assume that once a vesicle is attached to the microtubule with at least one motor protein, it is actively transported along the microtubule with a constant velocity, as it is also proposed by others [14]. The attachment of the vesicle by multiple motor proteins does not increase the vesicle speed, but only the attachment probability. The microtubule associated protein tau acts as a competitive inhibitor for kinesin binding. A vesicle that binds two active kinesin molecules can more easily overcome this competitive inhibition than a vesicle that only binds one active kinesin molecule.

We implement these observations into our model as a two-step process. We first calculate the fraction of anterograde (retrograde) vesicles that are bound to the microtubule based on the number of kinesin (dynein) receptors that are bound to the microtubule via kinesin (dynein) (we assume that all motor protein receptors are bound to a motor protein). Then we calculate the rate of membrane movement along the compartment under consideration of the vesicle speed and the length of the compartment.

Microtubules emerge from the TGN and TGN budded vesicles are loaded to the microtubules during the budding process [6]. Similarly, the growth cone contains microtubules within its central domain, some of which reach into the peripheral domain and are repelled by the actin treadmilling of the F-actin bundles. We therefore assume that TGN budded and endocytosed vesicles can immediately interact with the microtubules via their motor protein receptors and are transported into the NSC compartment via active microtubule-based transport.

Anterograde vesicles (with kinesin receptors that bind to active kinesin motor proteins) are first transported along the microtubules in the cell body cytoplasm, then along the microtubules in the NSC and finally arrive in the growth cone cytoplasm where they can fuse with the growth cone plasma membrane. Retrograde vesicles (with dynein receptors that bind to active dynein motor proteins) move the other way around. The three different compartments differ in the dissociation constants for kinesin and dynein that are influenced by the tau concentrations as described above.

Based on such compartment and motor protein specific dissociation constants we calculate the total number of motor protein receptors of one vesicle set that are bound to the microtubule in the compartment. Based on the average number of motor protein receptors per vesicle, we calculate the likelihood for each vesicle to be bound to the microtubule by at least one motor protein molecule using a binomial distribution. This likelihood equals the fraction of bound vesicles and can be used to calculate the total amount of membrane that is bound to and actively transported along the microtubule with a fixed velocity.

As already described, we do not track individual vesicles, but vesicle compartments within the individual compartments cell body cytoplasm, neurite shaft cytoplasm and growth cone cytosol. To model active vesicle transport along the microtubule, we estimate the flux of membranes in each vesicle compartment based on the time an individual vesicle would need to move along the neurite shaft cytoplasm. The transit time depends on the length of the microtubule in the particular compartment and the velocity of the vesicle. Under the steady-state assumption that all vesicles of one set in one compartment are uniformly distributed along the microtubules (i.e. the distance between traveling vesicles is always the same), we can estimate the frequency of vesicle arrival at the end of the neurite shaft cytoplasm by reversing the traveling time. This allows for the calculation of membrane flux from each vesicle compartment into its destination compartment, e.g. from the neurite shaft cytoplasm into the growth cone cytoplasm. We are aware of the fact that our approach causes forward, and backward vesicle movements based on changes in the neurite shaft cytoplasm length. Our vesicle transport approach assumes that changes in the length of the neurite shaft cytoplasm occur equally at every position, such as the whole neurite shaft cytoplasm would be stretched or compressed, so that the vesicles binding to it will also change their position. We nevertheless think, that this is a reasonable simplification allowing us to avoid tracking of individual vesicles, since, especially at later stages, the relative length changes of the neurite shaft cytoplasm should be small with regard to the vesicle velocity, as should be their influence on steady-state vesicle movement.

**Introduction of vesicle and target SNAREs and site-specific SNARE complex formation**

Vesicle fusion with the target (or donor) organelle is mediated via SNARE complex formation between Y-, V-, X- and U-SNAREs on the vesicle and their counterpart on the membrane of the target organelle as implemented in the original model [1]. SNARE-complex formation is mediated via tethering complexes that consist of cytoplasmic and/or peripheral membrane proteins [34, 35]. The fusion rate constant in the original model can be interpreted as a tethering rate constant. Anterograde vesicles bud from the TGN mediated by the coat protein B that has a high affinity for SNARE Y and SNARE V, retrograde vesicles bud from the growth cone mediated by coat protein A that has a high affinity for SNARE U and SNARE X. Consequently, anterograde vesicles enrich SNARE Y and SNARE V from the TGN and transport them to the growth cone plasma membrane, while retrograde vesicles enrich SNARE X and SNARE U from the growth cone membrane and transport them to the TGN, resulting in an asymmetric distribution of the two different SNARE sets at the two organelles. Consequently, vesicle fusion at the growth cone is mediated via SNARE complex formation between SNARE Y and SNARE V and vesicle fusion at the TGN via SNARE complex formation between SNARE X and SNARE U. To ensure that the next set of budding vesicles at the TGN can recruit Y- and/or V-SNAREs that allow their fusion with the growth cone membrane, at least one of the SNARE Y or SNARE V needs to be back transported to the TGN via retrograde vesicles. The same accounts for SNARE X and SNARE U in the opposite direction. Simplified, SNAREs can be separated into two sets of SNAREs, vesicle(v)- and target(t)-SNAREs [36]. We introduce such a separation and define that SNARE V and SNARE U are v-SNAREs and SNARE Y and SNARE X are t-SNAREs. By lowering the affinity of the t-SNAREs to the coat protein that mediates the budding of the vesicles that would bring the SNAREs back to the donor organelle, we ensure that mainly v-SNAREs are back transported, while t-SNAREs stay at the target organelle. Site specificity of the tethering machinery is achieved via different tethering rates for the two complementary sets of SNAREs, SNARE X and SNARE U complexes form with a higher rate constant at the TGN, while SNARE Y and SNARE V complexes form with a higher rate constant at the growth cone membrane.

**Growth of neurite shaft and diffusion block for transmembrane proteins**

The growth cone in our model has a size of 50 µm^2^ as it was experimentally documented for the growth cones of minor processes and growth cones of actively growing neurites [37]. Any membrane that is added to the growth cone that would exceed this size is de facto added to the neurite shaft compartment to increase its length. although the precise molecular mechanisms may involve multiple steps. In contrast, membrane proteins will always stay at the growth cone plasma membrane. We assume that high specialization of the growth cone membrane is mediated by intramembrane block that prevent the diffusion of the proteins into the neurite shaft. This assumption allows us to introduce a new unit, i.e. the neurite shaft membrane. Once the modeled growth cone reaches its experimentally determined size, net added membrane will be directed to the neurite shaft in the model. Similarly, membrane area that is removed from the growth cone plasma membrane will be first removed from the shaft membrane, since the size of the growth cone stays constant. In contrast, any transport components will be prevented from entering the neurite shaft membrane by the diffusion barriers to prevent an out dilution of transport components that would end vesicle transport in our model or demand an excess production of membrane proteins at the TGN.

**Production of membrane and transmembrane s at the TGN**

During neurite outgrowth new membrane needs to be synthesized in the cell body and transported to the growth cone tip. We assume that newly produced membrane is finally added to the TGN and from there transported to the growth cone tip based on experimental data [2, 3]. Since the length of NSC within the growing neurite shaft also increases during NOG, it will contain more vesicles, i.e. more membrane as well as trans membranous proteins. This loss of membrane and membrane proteins needs to be compensated by a continuous additional production of extra membrane lipids as well as protein proteins at the TGN. Therefore, we included a membrane lipid production rate and one production rate for each of the membrane proteins into our model.

**Vesicle transport model constraints**

Searching for combinations of transmembrane protein amounts and parameters that allow NOG at specified velocities, we defined model constraints that characterize physiological outgrowth. The model constraints referred to the rate of back transported membrane from the growth cone plasma membrane to the TGN and to the fraction of moving and stationary vesicles in the NSC, growth cone cytoplasm and cell body cytoplasm.

The complete membrane area of the growth cone is internalized within 30 - 60min [38]. Since we assume a growth cone surface area size of 50 um^2^ for the growth cone of a growing neurite [37], the endocytosis rate at the growth cone should be between ~0.4 and 0.8 μm^2^/min. Based on this estimation we assume that endocytosed retrograde vesicles should transport 0.5 μm^2^ membrane /min back to the TGN.

The observation that only a small fraction of the vesicles in the NSC are moving (9%) [1] suggests that the NSC functions as a reservoir or buffer that allows the adjustment the amount of membrane that fuses with the growth cone plasma membrane to the current demand. This assumption is supported by the fact that even after disruption of the TGN the neurite still grows for about 6h [19]. Furthermore, stop and go growth behavior of neurites [39, 40] favors the existence of a membrane buffer in the neurite shaft cytoplasm, since the adaptation of membrane delivery from the TGN to a switch in outgrowth velocity might take too long. Although our model simulates continuous steady growth of the neurite, we are including the membrane reservoir in the NSC that would allow such growth behavior. The aim of our model is the simulation of neurite outgrowth under the assumption that only 10% of the vesicles in the neurite shaft cytoplasm are actively transported towards the growth cone tip, while the other 90% function as a membrane reservoir that allows increased membrane mobilization in case of increased demand.

Similarly, we assume that the vesicles in the growth cone cytoplasm are the first source of additional membrane to increase NOG on demand. Therefore, we also assume that 10% of the vesicles in the growth cone cytoplasm are moving, i.e. fusing with the growth cone membrane which is in agreement with the observation that clusters of plasmalemma precursor vesicles (PPVs) persist in the growth cone with a half-life of at least 14 min (this suggests that 5% of the vesicles in the growth cone cytoplasm are moving) [41].

We think that there is no advantage of establishing such a pool of stationary retrograde vesicles in the neurite shaft cytoplasm. Therefore, we defined as two further model constraints that ~90% of retrograde vesicles in the neurite shaft cytoplasm and the cell body cytosol should be moving, i.e. either via dynein mediated active transport or SNARE-mediated fusion with the TGN.

**Identification of the dependency of average dynamic MT length and dynamic MT degradation rates on the effective tubulin concentration and GTP hydrolysis rate:**

To generate two formulas that describe the behavior of dynamic MTs that can be incorporated into our main model we simulated dynamic MT growth profiles under varying effective tubulin concentrations and GTP hydrolysis rates using the model of Margolin and co-workers [42]. We selected this model, since it offers a desirable balance between computational performance and biological detail. To estimate the length distribution of dynamic microtubules, we simulated the behavior of one microtubule (i.e. 13 interacting protofilaments) over 26,000 secs and calculated the frequency of all microtubule lengths over this time (in supplementary S2 Fig). We assume that the growth dynamics of all dynamic microtubules in the neurite are independent of each other. Based on this assumption the frequency of a certain microtubule length within this time period is also proportional to the frequency of a certain microtubule length among multiple microtubules at one timepoint. As a consequence, we estimate the length distribution of all dynamic microtubules based on the simulation of the growth and catastrophic behavior of one dynamic microtubule.

We used the model of Margolin et al to generate different length distributions of the microtubules in dependence of different effective tubulin concentrations. We calculated the microtubule length by averaging the length of the 13 protofilaments at each time point. The higher the effective tubulin concentration the longer the dynamic microtubules. Effective tubulin concentration is defined to be the concentration of free tubulin that is available for the incorporation into dynamic microtubules. For each effective tubulin concentration and GTP hydrolysis rate we determined the average length of dynamic MTs and developed a formula that describes the dependency of the average dynamic MT length on both variables (using third order polynomial fitting).

$Average Dynamic MT Length =-93.85+92.94x-752y-10.17x^{2}-13.45xy + 1159y^{2}+0.9806x^{3}-21.02x^{2}y+260.1{xy}^{2} -1588y^{3}.$

Where x is effective tubulin concentration and y is GTP hydrolysis rate.

The same dynamic MT growth profiles were used to identify the dependency of the dynamic MT degradation rate on the effective tubulin concentration. We assumed that a dynamic MT undergoes complete catastrophic breakdown, if its length is below 4 tubulin dimers. Based on this assumption, we counted how often the dynamic MT of each growth profile undergoes complete catastrophic breakdown. The dynamic MT degradation rate for each effective tubulin concentration was obtained by dividing this number by the simulation time. A power fitting method was used to obtain a formula that describes the dependence of the degradation rate on the effective tubulin concentration and the GTP hydrolysis rate (using third order polynomial fitting).

$$Degradation Rate=0.1521-0.08605 x+0.5545y+0.01416x^{2}-0.1517xy+0.2859y^{2}$$

$$-0.0006492x^{3}+0.007209x^{2}y+0.004233{xy}^{2}-0.1453y^{3}.$$

Where $x$ and $y$are effective tubulin concentration and GTP hydrolysis.

**Image Analysis:** Time lapsed images were acquired on the IN Cell Analyzer 2200. We used MetaMorph® software for image stitching of individual image tiles. Neurites were manually pseudo colored for quantification of individual neurites. Neurite lengths were quantified using the application module “neurite outgrowth” of MetaMorph®.

**Development of analytical solution for prediction of steady state dynamic relationships**

The development of the analytical solution starts with the consideration of the destination of the trafficking membrane (Fig 5(a)). Membrane that is incorporated into vesicles that bud from the TGN can be separated into 4 different membrane types. These membrane types should not be confused with the four-different vesicle sets in our model that distinguish the vesicles based on the organelle they budded from and the coat protein they budded with (Fig 1(b)). Membrane of the first type will be added to the growing neurite shaft cytoplasm as part of the neurite shaft cytoplasm reservoir for anterograde moving vesicles. Membrane of the second type will be added to the GC and from there to the growing neurite shaft. Membrane of the third and fourth types will also be added to the GC, but the fused membrane will be incorporated into endocytic retrograde vesicles. Similarly, the first membrane set, the third set will be added to the growing neurite shaft cytoplasm, though this set is much smaller due to an assumed nine-fold lower amount of stationary retrograde vesicles in the neurite shaft cytoplasm. The fourth membrane set will be back transported to the TGN, i.e. this set constitutes the cycling membrane between the TGN and the GC. For simplification, we assume in our solution that membranes transported in an anterograde manner is only transported as vesicles that bud from the TGN with coat protein B and retrogradely transported membranes are vesicles that bud from the GC with coat protein A.

The specification of an NOG velocity, the amount of cycling membrane and the percentages of stationary anterograde and retrograde vesicles in the neurite shaft cytoplasm allows the calculation of the fluxes of the 4 different membrane types.

**Type II membrane transport rate:**

$$Growth related k membrane production= \frac{NOG velocity}{2\pi\times radius of neurite} . (s1)$$

**Type IV membrane transport rate:**

$final back transport rate=cycling rate , (s2)$

$$a2 travel time new NSC=\frac{NSC length at start}{v_{d}} , (s3)$$

$$rate lost na2 NSC= \frac{final backward transport rate\times a2 travel time new NSC}{anticipated fraction of bound a2 vesicles in NSC} . (s4)$$

**Type III membrane transport rate:**

$$initial back transport rate=final backward transport rate+ rate lost na2 NSC, \left( S5 \right)$$

$final forward transport rate=initial back transport rate+ Growth related k membrane production , (S6)$

$$b1 travel time new NSC=\frac{NSC length at start}{v_{k}} , (S7)$$

$$rate lost nb1 NSC= \frac{final forward transport rate\times b1 travel time new NSC}{anticipated fraction of bound b1 vesicles in NSC} . (S8)$$

**Type I membrane transport rate:**

$$initial forward transport rate=final forward transport rate+ rate lost nb1 NSC , (S9)$$

$$rate lost surface area in NSC= rate lost nb1 NSC+ rate lost na2 NSC . (S10)$$

Based on these, the membrane production rate at the TGN as well as the budding rates at the TGN and GC can be calculated. The membrane production rate at the TGN is the sum of the fluxes of the first three membrane types, i.e. all membrane types that are not back transported to the TGN.

$$k membrane production rate= Growth related k membrane production$$

$+ rate lost surface area in NSC .$ $(S11)$

The budding rate at the TGN (or initial forward transport rate) is the sum of all 4 fluxes and the endocytosis rate (or initial backward transport rate) is the sum of the fluxes of the third and fourth types.

$$w_{G}^{B}= \frac{initial forward transport rate}{{cc1}_{G}\times s_{G}} , (S12)$$

$$w_{PM}^{A}= \frac{initial backward transport rate}{{cc2}_{PM}\times s_{PM}} , (S13)$$

$$w_{PM}^{B}= w_{G}^{B}\times factor for site specific budding, (S14)$$

$$w_{G}^{A}= w_{PM}^{A}\times factor for site specific budding. (S15)$$

The fluxes also allowed us to calculate the initial membrane surface areas in the cytoplasmic compartments.

$$b1 travel time NSC at start= \frac{NSC length at start}{v_{k}} , (S16)$$

$$n_{NSC}^{B_{G}}= \frac{final forward transport rate \times b1 travel time NSC at start}{anticipated fraction of bound b1 vesicles in NSC} , (S17)$$

$$n_{GCC}^{B_{G}}= \frac{final forward transport rate}{anticipated fraction of fusing B_{G} vesicles in GCC} , (S18)$$

$$a2 travel time NSC at start= \frac{NSC length at start}{v_{d}} , (S19)$$

$$n_{NSC}^{A_{PM}}= \frac{final backword transport rate \times a2 travel time NSC at start}{anticipated fraction of bound a2 vesicles in NSC} , (S20)$$

$$n_{CBC}^{A_{PM}}= \frac{final backword transport rate}{anticipated fraction of fusion a2 vesicles in CBC} . (S21)$$

Similarly, to the fraction of anterograde vesicles that are bound to the microtubule and therefore moving in the neurite shaft cytoplasm, we define the fractions of moving anterograde vesicles in the GC cytoplasm (i.e. vesicles that fuse with the GC membrane) and moving retrograde vesicles in the CB cytoplasm (i.e. vesicles that fuse with the TGN) (Table S4). The anticipated membrane fusion rate at the growth cone (or final forward transport rate) is equal to the sum of the fluxes of the second, third and fourth membrane types, the anticipated membrane fusion rate at the TGN (or final backward transport rate) is equal to the flux of the fourth membrane type. This allows the calculation of the number of v-SNAREs that are associated with each set of vesicles in both cytoplasmic compartments. We are looking for that amount of v-SNAREs V in the GC cytoplasm or U in the CB cytoplasm that allows that the fractions of fusing vesicles equal the anticipated fractions and that the total membrane fluxes equal the final forward and final backward membrane fluxes under a predefined amount of t-SNAREs Y at the TGN and X at the GC and pre-defined tethering rates.

$kappaXU_{pm}= kappaXU_{g}\times factor for site specific SNARE complex formation ,$ $(S22)$

$kappaYV_{g}= kappaYV_{pm}\times factor for site specific SNARE complex formation ,$ $(S23)$

$$YV SNARE complexes GCC PM= \frac{final forward transport rate}{anterograde vesicle surface area}$$

$$\times SNARE complex per vesicle fusion , (S24)$$

$${VV}_{GCC}^{B_{G}}=\frac{YV SNARE complexes GCC PM}{kappaYV_{pm}}\times{YY}_{pm} , (S25)$$

$$V_{GCC}^{B_{G}}=\frac{{VV}_{GCC}^{B_{G}}}{n_{GCC}^{B_{G}}} . (S26)$$

Vesicles that fuse with the TGN or the GC transmit their SNAREs to the target organelle. Consequently, the vesicles that enter the CB cytoplasm or GC cytoplasm from the NSC should contain the same number of SNAREs to keep the number of SNARE molecules in the cytoplasmic compartments’ constant. Additionally, v-SNAREs need to be back transported to the GC or the TGN to be available for the next set of budding vesicles. The fluxes for each v-SNARE can be associated to 4 different types in a similar manner as the membrane fluxes. Initial and final forward and backward fluxes can be calculated accordingly.

Similarly, to the v-SNAREs V, we calculate the corresponding parameter for v-SNARE U.

$$XU SNARE complexes CBC G= \frac{final backtransport rate}{anterograde vesicle surface area}$$

$\times SNARE complex per vesicle fusion$, $(S27)$

$${UU}_{CBC}^{A_{PM}}=\frac{XU SNARE complexes CBC G}{kappa{XU}_{G}}\times{XX}_{G} , (S28)$$

$$U_{CBC}^{A_{PM}}=\frac{{UU}_{CBC}^{A_{PM}}}{n_{CBC}^{A_{PM}}} , (S29)$$

${U\_backward=U}_{CBC}^{A_{PM}}$ , $(S30)$

$UU final backward=U\_backward\times final backtransport rate$, $(S31)$

$UU initial backward=U\_backward\times initial backtransport rate$, $(S32)$

$UU final forward=UU Initial backward$ , $(S33)$

$$U_{backward}=\frac{UU final forward}{final forward transport rate} , (S34)$$

$UU initial forward=initial forward transport rate \times U\_forward$. $(S35)$

SNAREs are recruited into the budding vesicles in a competitive manner, i.e. all 4 SNAREs compete with each other for the SNARE binding spots of the budding vesicles. The amounts of t-SNAREs Y and X at the GC and the TGN are predefined. Considering this we calculated the amount of v-SNAREs V and U at the TGN and GC that allow the calculated initial forward and backward v-SNARE fluxes.

$$Y_{PM}=\frac{{YY}_{PM}}{s_{PM}}, (S36)$$

$$X_{PM}=kxa \times\frac{Y_{PM}}{kya}, (S37)$$

$$cont_{PM}=1+\frac{Y_{PM}}{kya}+\frac{X_{PM}}{kxa}, (S38)$$

$bs=initial backtransport rate \times snare binding spot per vesicle area$ , $(S39)$

$$U_{PM}= \frac{UU initial backward \times cont_{PM}\times kua}{\left( bs-\left( UU initial backward+VV initial backward \right) \right)} , (S40)$$

$$V_{PM}= \frac{VV initial backward \times cont_{PM}\times kva}{\left( bs-\left( UU initial backward+VV initial backward \right) \right)} , (S41)$$

${UU}_{PM}=U_{PM} \times s_{PM}, (S42)$

$${XX}_{PM}=X_{PM} \times s_{PM}. (S43)$$

Though t-SNAREs have a higher dissociation constant for association with the vesicles than v-SNAREs they still are transported between the TGN and GC. We calculate their transport rates in a similar manner as for the vesicle SNAREs.

$snare saturation denom 2a=1+\frac{X_{PM}}{kxa}+\frac{U_{PM}}{kua}+ \frac{Y_{PM}}{kya}+ \frac{V_{PM}}{kva}$ , $(S44)$

$$sya2=\frac{snare binding spot per vesicle area\times\frac{Y_{PM}}{kya}}{snare saturation denom 2a} , (S45)$$

$YY initial backward=sya2\times initial backtransport rate$ $, (S46)$

$YY final backward=sya2\times final backtransport rate$ $, (S47)$

$$Y initial backward= \frac{YY initial backward}{initial backtransport rate} , (S48)$$

$YY final forward=YY initial backward$, $, (S49)$

$$Y forward= \frac{YY final forward}{final forward transport rate} , (S50)$$

$Y initial forward= Y forward\times initial forward transport rate$ . $, (S51)$

The 'consumption' of membrane proteins that are associated with the vesicles that are added to the NSC reservoir demands the continuous production of membrane proteins at the TGN. The protein production rates can be calculated by considering the concentration of each protein at anterograde and retrograde moving vesicles and the amount of membrane that will be added to the NSC reservoir as shown for the SNAREs V and Y

$$k VV production=rate lost nb1 NSC\times V forward+rate lost na2 NSC$$

$\times V backward, (S52)$

$$k YY production=rate lost nb1 NSC\times Y forward+rate lost na2 NSC$$

$\times Y backward$. $(S53)$

The concentration of the t-SNARE Y at the TGN is calculated via the following equations.

$X_{G}=\frac{{XX}_{G}}{s_{G}}$, $(S54)$

$${YY}_{G}=\max\left( 0, Y forward\times\frac{X_{G}}{kxb}\times kyb \times s_{G}- k YY production \right) . (S55)$$

TGN concentrations for the v-SNARE V and U are calculated in a similar manner as their concentrations at the GC.

t-SNARE X movements are calculated similarly as t-SNARE Y movements.

$snare saturation denom 1b=1+\frac{X_{G}}{kxb}+\frac{U_{G}}{kub}+ \frac{Y_{G}}{kyb}+ \frac{V_{G}}{kvb}$ , $(S56)$

$$sxb1= \frac{snare binding spot per vesicle area\times\frac{X_{G}}{kxb}}{snare saturation denom 1b} , (S57)$$

$X forward= sxb1 ,$ $(S58)$

$XX initial forward=X forward\times initial forward transport rate$ , $(S59)$

$XX final forward=X forward\times final forward transport rate ,$ $(S60)$

$XX initial backward=XX final forward$ , $(S61)$

$X backward= \frac{XX initial forward}{initial backward transport rate}$ , $(S62)$

$XX final backward=X backward\times final backward transport rate .$ $(S63)$

Production rates for SNAREs U and X are calculated.

SNARE X, U and V protein amounts at the TGN are calculated:

$$k UU production=rate lost nb1 NSC\times U forward+rate lost na2 NSC$$

$\times U backward, (S64)$

$$k XX production=rate lost nb1 NSC\times X forward+rate lost na2 NSC$$

$$\times X backward \left( S65 \right)$$

Protein for all vesicle membrane proteins in the different compartments are calculated:

${UU}_{G}=max(0, ({UU}_{G}-k UU production))$, $(S66)$

${XX}_{G}=max(0, ({XX}_{G}-k XX production))$ ,$(S67)$

${VV}_{G}=max(0, ({VV}_{G}-k VV production))$.$(S68)$

We calculated the amount of motor protein receptors that are associated with anterograde and retrograde vesicles in the neurite shaft cytoplasm that is necessary to allow the anticipated fraction of microtubule bound (i.e. moving) vesicles. This enabled the calculation of the motor protein receptors in the other cytoplasmic compartments and the amount of motor protein receptors at the TGN and GC as well as their production rates in a similar manner as we did it for the v-SNAREs.

$\left. \begin{aligned} {VV}_{CBC}^{B_{G}}= n_{CBC}^{B_{G}}\times V forward \\ {VV}_{NSC}^{B_{G}}=n_{NSC}^{B_{G}}\times V forward \\ {VV}_{GCC}^{B_{G}}= n_{GCC}^{B_{G}}\times V forward \\ {VV}_{GCC}^{A_{PM}}= n_{GCC}^{A_{PM}}\times V backward \\ {VV}_{NSC}^{A_{PM}} = n_{NSC}^{A_{PM}}\times V backward \\ {VV}_{CBC}^{A_{PM}}=n_{CBC}^{A_{PM}}\times V backward \end{aligned} \right\},$ $(S69)$

$\left. \begin{aligned} {UU}_{CBC}^{B_{G}}= n_{CBC}^{B_{G}}\times U forward \\ {UU}_{NSC}^{B_{G}}=n_{NSC}^{B_{G}}\times U forward \\ {UU}_{GCC}^{B_{G}}= n_{GCC}^{B_{G}}\times U forward \\ {UU}_{GCC}^{A_{PM}}= n_{GCC}^{A_{PM}}\times U backward \\ {UU}_{NSC}^{A_{PM}} = n_{NSC}^{A_{PM}}\times U backward \\ {UU}_{CBC}^{A_{PM}}= n_{CBC}^{A_{PM}}\times U backward \end{aligned} \right\},$ $(S70)$

$\left. \begin{aligned} {XX}_{CBC}^{B_{G}}= n_{CBC}^{B_{G}}\times X forward \\ {XX}_{NSC}^{B_{G}}=n_{NSC}^{B_{G}}\times X forward \\ {XX}_{GCC}^{B_{G}}= n_{GCC}^{B_{G}}\times X forward \\ {XX}_{GCC}^{A_{PM}}= n_{GCC}^{A_{PM}}\times X backward \\ {XX}_{NSC}^{A_{PM}} = n_{NSC}^{A_{PM}}\times X backward \\ {XX}_{CBC}^{A_{PM}}= n_{CBC}^{A_{PM}}\times X backward \end{aligned} \right\},$ $(S71)$

$\left. \begin{aligned} {YY}_{CBC}^{B_{G}}= n_{CBC}^{B_{G}}\times Y forward \\ {YY}_{NSC}^{B_{G}}=n_{NSC}^{B_{G}}\times Y forward \\ {YY}_{GCC}^{B_{G}}= n_{GCC}^{B_{G}}\times Y forward \\ {YY}_{GCC}^{A_{PM}}= n_{GCC}^{A_{PM}}\times Y backward \\ {YY}_{NSC}^{A_{PM}} = n_{NSC}^{A_{PM}}\times Y backward \\ {YY}_{CBC}^{A_{PM}}=n_{CBC}^{A_{PM}}\times Y backward \end{aligned} \right\}.$ $(S72)$

Finally, we calculated the fluxes for the other stationary proteins, i.e. recruitment factors 1 and 2 as well as the initial protein amounts in the different cytoplasmic compartments.

$${KK}_{NSC}^{B_{G}}= \frac{log(1-anticipated fraction of bound vesicles in NSC)}{log(1-fraction bound kk a1b1 NSC)} , (S73)$$

$$K_{NSC}^{B_{G}}= \frac{{KK}_{NSC}^{B_{G}}}{anterograde vesicles surface area} , (S74)$$

$K forward= K_{NSC}^{B_{G}},$ $(S75)$

$KK initial forward=K forward\times initial forward transport rate ,$ $(S76)$

$KK final forward=K forward\times final forward transport rate ,$ $(S77)$

$KK initial backward=KK final forward ,$ $(S78)$

$$K backward= \frac{KK initial backward}{initial backward traport rate} , (S79)$$

$$MBSPVA= motor binding spots per vesicles area,$$

$$K_{G}=\frac{kkb\times KK initial forward}{initial forward traport rate\times MBSPVA-KK initial forward}, (S80)$$

$$K_{PM}=\frac{kka\times KK initial backward}{initial backward traport rate\times MBSPVA-KK initial backward} , (S81)$$

$${KK}_{G}=K_{G} \times s_{G} , (S82)$$

$${KK}_{PM}=K_{PM} \times s_{PM}, (S83)$$

${KK}_{CBC}^{B_{G}}=k forward\times n_{CBC}^{B_{G}},$ $(S84)$

$${KK}_{NSC}^{B_{G}}=k forward\times n_{NSC}^{B_{G}}, (S85)$$

${KK}_{GCC}^{B_{G}}=k forward\times n_{GCC}^{B_{G}},$ $(S86)$

${KK}_{CBC}^{B_{PM}}=k backward\times n_{CBC}^{A_{PM}},$ $(S87)$

${KK}_{NSC}^{B_{PM}}=k backward\times n_{NSC}^{A_{PM}}$,$(S88)$

$${KK}_{GCC}^{B_{PM}}=k backward\times n_{GCC}^{A_{PM}}, (S89)$$

$$k KK production =rate lost nb1 NSC\times K forward +rate lost na2 NSC\times K backward, (S90)$$

$${DD}_{NSC}^{B_{G}}= \frac{log(1-anticipated fraction of bound vesicles in NSC)}{log(1-fraction bound dd a2b2 NSC)} , (S91)$$

$$D_{NSC}^{A_{PM}}= \frac{{DD}_{NSC}^{A_{PM}}}{anterograde vesicles surface area} , (S92)$$

$$D backward= {DD}_{NSC}^{A_{PM}}, (S93)$$

$DD initial backward=D backward\times initial backward transport rate , (S94)$

$DD final forward= DD initial backward ,$ $(S95)$

$DD initial forward=D forward\times initial forward transport rate, (S96)$

$$D_{G=}=\frac{kdb\times DD initial forward}{initial forward traport rate\times MBSPVA-DD initial forward} , (S97)$$

$$K_{PM}=\frac{kda\times DD initial backward}{initial backward traport rate\times MBSPVA-DD initial backward} , (S98)$$

${DD}_{G}=D_{G} \times s_{G} , (S99)$

$${DD}_{PM}=D_{PM} \times s_{PM}, (S100)$$

$${DD}_{CBC}^{B_{G}}=D forward\times n_{CBC}^{B_{G}}, (S101)$$

$${DD}_{NSC}^{B_{G}}=D forward\times n_{NSC}^{B_{G}}, (S102)$$

${DD}_{GCC}^{B_{G}}=D forward\times n_{GCC}^{B_{G}}, (S103)$

$${DD}_{CBC}^{A_{PM}}=D backward\times n_{CBC}^{A_{PM}}, (S104)$$

$${DD}_{NSC}^{A_{PM}}=D backward\times n_{NSC}^{A_{PM}}, (S105)$$

$${DD}_{GCC}^{A_{PM}}=D backward\times n_{GCC}^{A_{PM}}, (S106)$$

$k DD production=rate lost nb1 NSC\times D forward+rate lost na2 NSC$ $\times D backward , (S107)$

${C1}_{G}= \frac{{CC1}_{G}}{s_{G}}$ , $(S108)$

$CBSPVA= cargo binding spots per vesicl area$.

$$sc1b1= \frac{CBSPVA \times\frac{{C1}_{G}}{kc1b}}{\left( 1+\frac{{C1}_{G}}{kc1b} \right)} , (S109)$$

$$C1 forward= sc1b1, (S110)$$

$$CC1 final forward= C1 forward \times final forward transport rate, (S111)$$

$CC1 initial backward= CC1 final forward$, $(S112)$

$$C1 backward= \frac{CC1 initial backward}{initial backtransport rate} , (S113)$$

$${C1}_{PM}= \frac{kc1a\times CC1 initial backward}{initial backward traport rate\times MBSPVA-CC1 initial backward} , (S114)$$

${CC1}_{PM}={C1}_{PM} \times s_{PM}, (S115)$

$${CC1}_{CBC}^{B_{G}}=C1 forward\times n_{CBC}^{B_{G}}, (S116)$$

$${CC1}_{NSC}^{B_{G}}=C1 forward\times n_{NSC}^{B_{G}}, (S117)$$

${CC1}_{GCC}^{B_{G}}=C1 forward\times n_{GCC}^{B_{G}}, (S118)$

${CC1}_{CBC}^{A_{PM}}=C1 backward\times n_{CBC}^{A_{PM}}, \left( S119 \right)$

$${CC1}_{NSC}^{A_{PM}}=C1 backward\times n_{NSC}^{A_{PM}}, (S120)$$

${CC1}_{GCC}^{A_{PM}}=C1 backward\times n_{GCC}^{A_{PM}}, (S121)$

$$k CC1 production = rate lost nb1 NSC\times C1 forward +rate lost na2 NSC$$

$\times C1 backward , (S122)$

$${C2}_{PM}= \frac{{CC2}_{PM}}{s_{PM}} , (S123)$$

$$sc2a2= \frac{CBSPVA \times\frac{{C2}_{PM}}{kc2a}}{\left( 1+\frac{{C2}_{PM}}{kc2a} \right)} , (S124)$$

$C2 backward= sc2a2 , (S125)$

$$CC2 initial backward= C2 backward \times initial back transport rate , (S126)$$

$CC2 final forward= CC2 initial backward, (S127)$

$C2 forkward= \frac{CC2 final forward}{final forward transport rate} , \left( S128 \right)$

$C2 initial forkward= C2 forward \times initial forward transport rate , (S129)$

$${C2}_{G}= \frac{kc2b\times CC2 initial forward}{initial forward traport rate\times MBSPVA-CC2 initial forward} , (S130)$$

${CC2}_{G}={C2}_{G} \times s_{G} , (S131)$

${CC2}_{CBC}^{B_{G}}=C2 forward\times n_{CBC}^{B_{G}}, (S132)$

$${CC2}_{NSC}^{B_{G}}=C2 forward\times n_{NSC}^{B_{G}}, (S133)$$

${CC2}_{GCC}^{B_{G}}=C2 forward\times n_{GCC}^{B_{G}},$ ($S134)$

${CC2}_{CBC}^{A_{PM}}=C2 backward\times n_{CBC}^{A_{PM}},$ $(S135)$

${CC2}_{NSC}^{A_{PM}}=C2 backward\times n_{NSC}^{A_{PM}},$ $(S136)$

$${CC2}_{GCC}^{A_{PM}}=C2 backward\times n_{GCC}^{A_{PM}}, (S137)$$

$$k CC2 production= rate lost nb1 NSC\times C2 forward+rate lost na2 NSC$$

$\times C2 backward . (S138)$

For the calculation of the rates that determine MT growth with a fixed velocity of choice we specified the effective tubulin concentration (that determines the length of the dynamic MTs and the degradation rate of dynamic MTs) and the length of the dynamic MTs. Based on these values the nucleation rate for dynamic MTs and the conversion rate of dynamic into stable MTs were calculated.

$$NOG velocity= MTB growth velocity=\frac{Length increase of stable\times MTs}{MTs per crossection} , (S139)$$

$$Number dynamic MTs= \frac{Total dyn MTs length}{(Average dynamic MT length)} , (S140)$$

$$stablization rate= \frac{Length increase of stable MTs}{(Average dynamic MT length \times Number dynamic MTs)} , (S141)$$

$$Nucleation rate =Stabilization rate \times Total number dynamic MTs$$

$$+Degradation rate\times Total number dynamic MTs. (S142)$$

**Validation of predicted kinetic parameters at steady state**

For each anticipated velocity (0, 2.5, 5, 7.5, 10, 12.5, 15, 17.5, 20 μm/h) we selected five sets of kinetic parameters that contained different combinations of predicted tethering rates and v-SNARE V concentrations. We numerically simulated NOG using these sets of parameters for 5000 minutes (~3.5 days). Every 500 minutes we documented the current growth velocity, cycling membrane, TGN size, percentages of the moving B_G_ vesicles in the NSC and GCC and the percentages of moving A_PM_ vesicles in the NSC and CBC. For each of these values, we calculated one average value and standard deviation for each anticipated velocity and compared the results to the pre-defined model constraints.

**Systematic analysis of SCP-relationships**

We used our analytical prediction of steady state dynamics to investigate relationships between Level-2 and Level-3 sibling SCPs. To investigate the relationships between Level-3 SCP sibling we selected one kinetic parameter of each Level-3 SCP. We predefined the kinetic parameter of one Level-3 SCP and used the analytical prediction to find the matching kinetic parameter of its sibling Level-3 SCP that allows NOG with a given velocity and without violation of the model constraints.

**Primary neuronal preparations**

All protocols comply with the IACUC at the Icahn School of Medicine at Mount Sinai. P1 rat cortical neurons were prepared from Long-Evans rat pups of both sexes. Cortices were dissected from P1 pups and we removed the meninges in plain Neurobasal-A (NB) on ice using aseptic techniques. The cortices were incubated twice at 37^o^C with 0.5mg/ml papain (Sigma) in plain NB for 30mins each, during the second incubation we added 50µg/ml DNaseI (Sigma). We triturated the tissue and strained them through a 70micron cell strainer. For the gradient to enrich for neuronal cultures, cell suspensions were layered on an Optiprep density gradient (Sigma) and centrifuged at RT at 1900xg for 15mins with the brake off. We used 6 cortices per gradient preparation. The purified neurons were collected into fresh NB supplemented with B27, L-glutamine and Penicillin-streptomycin (NB supplemented media) and centrifuged at RT at 1000g for 5mins to remove all residual Optiprep. The pellet was resuspended in 200µls of NB supplemented media for seeding into the microfluidic chambers.

**Neurite outgrowth assay**

To document the effect of siRNA ablation on neurite outgrowth, we plated primary cortical rat neurons in microfluidic chambers and transfected them with siRNA against selected genes. After an initial incubation period of about 48h that allowed for degradation of residual proteins, we used axotonomy to reset all neurites (in control and siRNA treated chambers) to the same outgrowth position. After a second incubation period of 48h that allowed outgrowth under knockdown conditions, we stopped the experiment and compared outgrowth characteristics of treated and control samples.

Microfluidic chambers with a 150µm micro-groove (Xona Microfluidics) were sterilized by incubating in 70% ethanol for 2 minutes. The chambers were removed from the ethanol and allowed to dry under a sterile tissue culture hood. The chambers with a pair of sterile forceps were gently placed on PLL-coated 50 mm glass bottom dishes (MatTek Corp.). With gentle force we made sure the silicone of the microfluidic chambers was securely on the glass of the dishes. First, we pre-loaded the neurite-growing compartment of the chambers with 300 µls of NB supplemented media. Using a 20 µl micro pipettor we seeded 15µls of the neuronal preparation into the compartment for the neuronal cell bodies. We placed the microfluidic chambers containing the neurons into a 37^o^C incubator with 5% CO_2_ for 20 mins, to allow the neurons to adhere to the surface and ensure a monolayer of cells in the compartment. Then we gently filled the rest of the neuronal cell bodies compartment with 300µls of NB supplemented media and returned to the 37^o^C incubator.

We custom made siRNA pools for the Vamp7 (CCAUCAUCGUCGUUGUAUC, GGAUUGU GUAUCUUUGCAU, UCAUGGCAAUUAUUUGUUU, CCAUGAACAGUGAGUUUUC) and Mtcl1 (GUGUUUUAUUUCAUGCUUC, UCAGAGUGUUCUAAGUUAA, CCCUGAAGC AGAACAUUUU, GCUUUGUCCCUGGAUGAUG) genes, as well as scrambled sequence for controls (Dharmacon). 90 min after plating the cortical neurons in the chambers we removed the media from the neuronal cell bodies chambers and added 150 µls of 1 µM siRNA in Dharmacon transfection media (accell siRNA delivery media) and incubated overnight. The next day the transfection reagent was replaced with NB supplemented media. To eliminate the basal neurite outgrowth not attributable to the siRNAs, we axotomized the neurites 48 h after treating with siRNA. Axotomy was performed by aspirating 2 times the neurite growing compartments with plain NB. We confirmed the neurites were severed under a light microscope and then filled the compartment with NB supplemented media and allowed the neurites to grow an additional 48 h. At this point both compartments were fixed with 4% paraformaldehyde. We immunostained both compartments using a monoclonal antibody anti-βIII tubulin (Tuj1; Covance) and Alexa Fluor 488-conjugated anti-mouse IgG (Invitrogen). For quantification of neurite length, we imaged on a LSM 880 confocal microscope (20x, zoom ins: 63x). We used Image J software using the Plot Profile function to analyze total neurite outgrowth from the microgroove edge and graphed our data using GraphPad Prism. Dystrophic bulbs were counted manual along the entire microgroove of the microfluidic chamber.

**Quantitative real-time PCR**. Knock down efficiency was validated by quantitative reverse transcription-PCR (Q-RT-PCR). Total RNA was extracted using Invitrogen™ TRIzol™ Reagent, the sample was spun at 12x10^3^ RPM at 4ᵒC for 10 minutes, the supernatant collected and mixed with 200uL of chloroform. The solution was spun at 12x10^3^ RPM at 4ᵒC for 10 minutes and the clear supernatant was transferred to a tube containing 500 ul of Isopropanol. The solution was mixed and incubated for 20 minutes at room temperature. The tubes were spun down spun at 14.8x10^3^ RPM at 4ᵒC for 10 minutes. The supernatant was discarded, and the pellet was then resuspended 75% Ethanol. The tubes were spun down at 7.5x10^3^ RPM for 10 minutes. This step was repeated twice. The supernatant was discarded, and the pellet was resuspended in 30ul of UltraPure™ DNase/RNase-Free Distilled Water. Applied Biosystems™ High-Capacity RNA-to-cDNA™ Kit was used to acquire cDNA. Equal amounts of total RNA (0.25µg) were used as template. Gene expression of Vamp7 and MTCL1 in rat primary cortical neurons was confirmed by using RT-PCR using selected gene-specific primers: GAPDH 5’-TGCGACTTCAACAGCAAC-3’ and 5’-CTTGCTCAGTGTCCTTGC-3’’, VAMP7 Rat 5’-GTTGCCAGGGGAACCACTAT-3’, 5’-GCCAGAACGCTCGAAAACTC-3’’ [43], and MTCL1 Rat 5’-ACCGGACAGATGGAACAGAC-3’, 5’-GTCTTCACGAAGCTCCGAAC-3’’. Applied Biosystems™ Power SYBR™ Green PCR Master Mix was added to triplicate samples of 10ng/µl cDNA and 200nM of each primer. The RT-PCR was performed using Applied Biosystems™ 7500 Real-Time PCR System as follows one cycle of 95ᵒC for 10 minutes to activate polymerase, and 40 cycles of denaturing at 95ᵒC for 15 seconds followed by annealing/extension at 60ᵒC for 1 minute. Data was analyzed using the 2 ^ΔΔCT^ method [44, 45].

**References:**

1. Heinrich R, Rapoport TA. Generation of nonidentical compartments in vesicle transport systems. The Journal of Cell Biology. 2005; 168: 271-280.
2. Gracias NG, Shirkey-Son NJ, Hengst U. Local translation of TC10 is required for membrane expansion during axon outgrowth. Nature Communications. 2014; 5: 3506.
3. Wang T, Liu Y, Xu XH, Deng CY, Wu KY, Zhu J, et al. Lgl1 activation of rab10 promotes axonal membrane trafficking underlying neuronal polarization. Developmental Cell. 2011; 21: 431-444.
4. Nakazawa H, Sada T, Toriyama M, Tago K, Sugiura T, Fukuda M, et al. Rab33a mediates anterograde vesicle transport for membrane exocytosis and axon outgrowth. The Journal of Neuroscience : the Official Journal of the Society for Neuroscience. 2012; 32: 12712-12725.
5. Pfenninger KH. Plasma membrane expansion: a neuron's Herculean task. Nature Reviews Neuroscience. 2009; 10: 251-261.
6. Burgo A, Proux-Gillardeaux V, Sotirakis E, Bun P, Casano A, Verraes A, et al. A molecular network for the transport of the TI-VAMP/VAMP7 vesicles from cell center to periphery. Developmental Cell. 2012; 23: 166-180.
7. Vale RD, Funatsu T, Pierce DW, Romberg L, Harada Y, Yanagida T. Direct observation of single kinesin molecules moving along microtubules. Nature. 1996; 380: 451-453.
8. Carter NJ, Cross RA. Mechanics of the kinesin step. Nature. 2005; 435: 308-312.
9. Dixit R, Ross JL, Goldman YE, Holzbaur EL. Differential regulation of dynein and kinesin motor proteins by tau. Science. 2008; 319: 1086-1089.
10. Ebneth A, Godemann R, Stamer K, Illenberger S, Trinczek B, Mandelkow E. Overexpression of tau protein inhibits kinesin-dependent trafficking of vesicles, mitochondria, and endoplasmic reticulum: implications for Alzheimer's disease. The Journal of Cell Biology. 1998; 143: 777-794.
11. Hagiwara H, Yorifuji H, Sato-Yoshitake R, Hirokawa N. Competition between motor molecules (kinesin and cytoplasmic dynein) and fibrous microtubule-associated proteins in binding to microtubules. The Journal of Biological Chemistry. 1994; 269: 3581-3589.
12. LaPointe NE, Morfini G, Pigino G, Gaisina IN, Kozikowski AP, Binder LI, at el. The amino terminus of tau inhibits kinesin-dependent axonal transport: implications for filament toxicity. Journal of neuroscience research. 2009; 87: 440-451.
13. Seitz A, Kojima H, Oiwa K, Mandelkow EM, Song YH, Mandelkow E. Single-molecule investigation of the interference between kinesin, tau and MAP2c. The EMBO Journal. 2002; 21: 4896-4905.
14. Vershinin M, Carter BC, Razafsky DS, King SJ, Gross SP. Multiple-motor based transport and its regulation by Tau. Proceedings of the National Academy of Sciences of the United States of America. 2007; 104: 87-92.
15. Black MM, Slaughter T, Moshiach S, Obrocka M, Fischer I. (1996). Tau is enriched on dynamic microtubules in the distal region of growing axons. The Journal of Neuroscience: the Official Journal of the Society for Neuroscience. 1996; 16: 3601-3619.
16. Kempf M, Clement A, Faissner A, Lee G, Brandt R. Tau binds to the distal axon early in development of polarity in a microtubule- and microfilament-dependent manner. The Journal of neuroscience : the official journal of the Society for Neuroscience. 1996; 16: 5583-5592.
17. Mandell JW, Banker GA. A spatial gradient of tau protein phosphorylation in nascent axons. The Journal of neuroscience : the official journal of the Society for Neuroscience. 1996*;*16: 5727-5740.
18. Ahmed WW, Saif TA. Active transport of vesicles in neurons is modulated by mechanical tension. Scientific Reports. 2014; 4: 4481.
19. Prager-Khoutorsky M, Spira ME. Neurite retraction and regrowth regulated by membrane retrieval, membrane supply, and actin dynamics. Brain research. 2009; 1251: 65-79.
20. Fu, MM, Nirschl JJ, Holzbaur EL. LC3 binding to the scaffolding protein JIP1 regulates processive dynein-driven transport of autophagosomes. Developmental cell. 2014; 29: 577-590.
21. Maday S, Wallace KE, Holzbaur EL. Autophagosomes initiate distally and mature during transport toward the cell soma in primary neurons. The Journal of cell biology. 2012; 196: 407-417.
22. Mada S, Twelvetrees AE, Moughamian AJ, Holzbaur EL. Axonal transport: cargo-specific mechanisms of motility and regulation. Neuron. 2014; 84: 292-309.
23. Prevo B, Mangeol P, Oswald F, Scholey JM, Peterman EJ. Functional differentiation of cooperating kinesin-2 motors orchestrates cargo import and transport in C. elegans cilia. Nature Cell Biology. 2015; 17: 1536-1545.
24. Encalada SE, Szpankowski L, Xia CH, Goldstein LS. Stable kinesin and dynein assemblies drive the axonal transport of mammalian prion protein vesicles. Cell. 2011; 144: 551-565.
25. Twelvetrees AE, Pernigo S, Sanger A, Guedes-Dias P, Schiavo G, Steiner RA, et al. The Dynamic Localization of Cytoplasmic Dynein in Neurons Is Driven by Kinesin-1. Neuron. 2016; 90: 1000-1015.
26. Godlee C, Kaksonen M. Review series: From uncertain beginnings: initiation mechanisms of clathrin-mediated endocytosis. The Journal of Cell Biology. 2013; 203*:* 717-725.
27. Lajoie P, Nabi IR. Regulation of raft-dependent endocytosis. Journal of Cellular and Molecular Medicine. 2007; 11: 644-653.
28. Mettlen M, Danuser G. Imaging and modeling the dynamics of clathrin-mediated endocytosis. Cold Spring Harbor Perspectives in Biology. 2014; 6: a017038.
29. Kienzle C, von Blume J. Secretory cargo sorting at the trans-Golgi network. Trends in Cell Biology. 2014; 24: 584-593.
30. Kim K. Cargo trafficking from the trans-Golgi network towards the endosome. Biology of the cell. 2016; 108*:* 205-218.
31. Lord C, Bhandari D, Menon S, Ghassemian M, Nycz D, Hay J, et al. Sequential interactions with Sec23 control the direction of vesicle traffic. Nature. 2011; 473: 181-186.
32. Collins A, Warrington A, Taylor KA, Svitkina T. Structural organization of the actin cytoskeleton at sites of clathrin-mediated endocytosis. Current Biology. 2011; 21: 1167-1175.
33. Lasek RJ, Garner JA, Brady ST. Axonal transport of the cytoplasmic matrix. The Journal of Cell Biology. 1984; 99: 212s-221s.
34. Dubuke ML, Munson M. The Secret Life of Tethers: The Role of Tethering Factors in SNARE Complex Regulation. Frontiers in cell and developmental biology. 2016; 4: 42.
35. Hong W, Lev S. Tethering the assembly of SNARE complexes. Trends in cell biology. 2014; 24*:* 35-43.
36. Jahn R, Scheller RH. SNAREs--engines for membrane fusion. Nature reviews Molecular cell biology. 2006; 7: 631-643.
37. Kunda P, Paglini G, Quiroga S, Kosik K, Caceres A. Evidence for the involvement of Tiam1 in axon formation. J Neurosci. 2001; 21*:* 2361-2372.
38. Diefenbach TJ, Guthrie PB, Stier H, Billups B, Kater SB. Membrane recycling in the neuronal growth cone revealed by FM1-43 labeling. The Journal of Neuroscience : the Official Journal of the Society for Neuroscience. 1999; 19: 9436-9444.
39. McKerracher L, Rosen KM. MAG, myelin and overcoming growth inhibition in the CNS. Frontiers in Molecular Neuroscience. 2015; 8: 51.
40. Shen Y. Traffic lights for axon growth: proteoglycans and their neuronal receptors. Neural Regeneration Research. 2014*;* 9: 356-361.
41. Pfenninger KH, Laurino L, Peretti D, Wang X, Rosso S, Morfini G, et al. Regulation of membrane expansion at the nerve growth cone. J Cell Sci. 2003; 116: 1209-1217.
42. Margolin G, Gregoretti IV, Cickovski TM, Li C, Shi W, Alber MS, et al. The mechanisms of microtubule catastrophe and rescue: implications from analysis of a dimer-scale computational model. Mol Biol Cell. 2012; 23: 642-656.
43. Szalinski CM, Labilloy A, Bruns JR, Weisz OA. VAMP7 modulates ciliary biogenesis in kidney cells. Neural regeneration research *9*, 356-361. Plos One. 2014; 9: e86425.
44. Livak KJ, Schmittgen TD. Analysis of Relative Gene Expression Data Using Real-Time Quantitative PCR and the 2^−ΔΔCT^ Method. Methods. 2001; 25: 402-408.
45. [Cao Z](https://www.ncbi.nlm.nih.gov/pubmed/?term=Cao%20Z%5BAuthor%5D&cauthor=true&cauthor_uid=16707807), [Gao Y](https://www.ncbi.nlm.nih.gov/pubmed/?term=Gao%20Y%5BAuthor%5D&cauthor=true&cauthor_uid=16707807), [Bryson JB](https://www.ncbi.nlm.nih.gov/pubmed/?term=Bryson%20JB%5BAuthor%5D&cauthor=true&cauthor_uid=16707807), [Hou J](https://www.ncbi.nlm.nih.gov/pubmed/?term=Hou%20J%5BAuthor%5D&cauthor=true&cauthor_uid=16707807), [Chaudhry N](https://www.ncbi.nlm.nih.gov/pubmed/?term=Chaudhry%20N%5BAuthor%5D&cauthor=true&cauthor_uid=16707807), [Siddiq M](https://www.ncbi.nlm.nih.gov/pubmed/?term=Siddiq%20M%5BAuthor%5D&cauthor=true&cauthor_uid=16707807), et al. The Cytokine Interleukin-6 Is Sufficient But Not Necessary to Mimicthe Peripheral Conditioning Lesion Effecton Axonal Growth. [J Neurosci.](https://www.ncbi.nlm.nih.gov/pubmed/16707807) 2006; 26: 5565-5573.
46. Ren Y, Suter DM. Increase in Growth Cone Size Correlates with Decrease in Neurite Growth Rate. Neural Plasticity. 2016*;* 3497901.
47. Yu W, Baas PW. Changes in microtubule number and length during axon differentiation. The Journal of Neuroscience: The Official Journal of the Society for Neuroscience. 1994; 14: 2818-2829.
48. Fadic R, Vergara J, Alvarez J. Microtubules and caliber of central and peripheral processes of sensory axons. J Comp Neurol. 1985; 236: 258-264.
49. Harris KM, Stevens JK. Dendritic spines of CA 1 pyramidal cells in the rat hippocampus: serial electron microscopy with reference to their biophysical characteristics. J Neurosci. 1989; 9*:* 2982-2997.
50. Beller JA, Kulengowski B, Kobraei EM, Curinga G, Calulot CM, Bahrami, A, et al. Comparison of sensory neuron growth cone and filopodial responses to structurally diverse aggrecan variants, in vitro. Exp Neurol. 2013; 247: 143-157.
51. King SJ, Schroer TA. Dynactin increases the processivity of the cytoplasmic dynein motor. Nat Cell Biol. 2000; 2: 20-24.
52. Nishiura M, Kon T, Shiroguchi K, Ohkura R, Shima T, Toyoshima YY, et al. A single-headed recombinant fragment of Dictyostelium cytoplasmic dynein can drive the robust sliding of microtubules. J Biol Chem. 2004; 279: 22799-22802.
53. Zhang B, Koh YH, Beckstead RB, Budnik V, Ganetzky B, Bellen HJ. Synaptic vesicle size and number are regulated by a clathrin adaptor protein required for endocytosis. Neuron. 1998; 21: 1465-1475.
